# The Effects of Pretreated and Fermented Corn Stalks on Growth Performance, Nutrient Digestion, Intestinal Structure and Function, and Immune Function in New Zealand Rabbits

**DOI:** 10.3390/ani15121737

**Published:** 2025-06-12

**Authors:** Xuying Jia, Yaohao Dun, Guoqi Xiang, Shuai Wang, Heng Zhang, Wen Zhou, Yingjun Li, Yunxiang Liang

**Affiliations:** 1 National Key Laboratory of Agricultural Microbiology, College of Life Science and Technology, Huazhong Agricultural University, Wuhan 430070, China; jiaxy@webmail.hzau.edu.cn (X.J.); dunyaohao@mail.hzau.edu.cn (Y.D.);; 2 Department of Animal Nutrition and Feed Science, College of Animal Science and Technology, Huazhong Agricultural University, Wuhan 430070, China; 3 Green Chemical Reaction Engineering, Engineering and Technology Institute Groningen (ENTEG), University of Groningen, Nijenborgh 4, 9747 AG Groningen, The Netherlands; 4 Cooperative Innovation Center of Industrial Fermentation (Ministry of Education & Hubei Province), Hubei University of Technology, Wuhan 430068, China

**Keywords:** fermented corn straw, corn substitutes, intestinal digestion and absorption, metabolome and microbial diversity, New Zealand rabbit

## Abstract

This study investigates the efficacy of fermented corn straw as a viable corn substitute in rabbit diets, evaluating its impact on growth performance, intestinal health, cecal microbiota, and metabolite profiles to determine its potential for addressing corn shortages in animal husbandry. Over 35 days, 120 New Zealand rabbits were assigned to four treatments: (i) 12% corn (C100), (ii) 6% corn + 6% fermented straw (FS50), (iii) 12% fermented straw (FS100), and (iv) 6% corn + 6% dry straw (DS50). Fermented straw enhanced the rabbits’ average daily feed intake (ADI) and average daily gain (ADG) and elevated cecal cellulase activity. It also downregulated *TLR4* in the jejunum mucosa, upregulated MUC2 in the ileum mucosa, strengthened the intestinal barrier, and reduced the diarrhea index and incidence in weaned rabbits. Specific microbial families influenced amino acid and phospholipid concentrations, altering the cecal metabolic environment. In summary, replacing corn with fermented corn straw in rabbit diets significantly boosts ADG and ADI, potentially lowers the feed-to-gain ratio, and enhances cecal microbiota and metabolite profiles without compromising growth performance.

## Simple Summary

This study explores the potential of pretreated and fermented corn stalks as a sustainable alternative to corn in the diets of New Zealand rabbits. It addresses a critical issue in livestock farming by identifying a novel feed source that can reduce dependency on corn, lower feed costs, and enhance the sustainability of animal husbandry. The research employs a robust sample size of 120 rabbits and examines multiple physiological parameters. It integrates multi-omics data, including microbiome and metabolome analyses, to provide a comprehensive understanding of the effects of fermented corn stalks on animal health and performance. Additionally, we incorporate detailed analyses of animal nutrition, histology, and metabolic pathways. Ethical approval is thoroughly documented, and the tables and figures are clear and well-labeled, facilitating the interpretation of the results.

## 1. Introduction

Corn is the main raw material in animal feed and is extremely important in animal husbandry. However, climate change will likely negatively affect the global food supply. To meet the ongoing demand for livestock products, it is necessary to develop new raw materials to replace corn to reduce the instability of the supply chain caused by excessive dependence on corn [1,2]. In recent years, an increasing number of studies have focused on replacing corn with different raw materials. With the increasing demand of global consumers for healthy food, rabbit meat is gradually favored because of its low fat and high protein characteristics [3], and the development of cold chain logistics technology makes rabbit meat better meet the needs of global consumers [4]. The annual breeding population of domestic rabbits worldwide has exceeded 1.5 billion, of which meat rabbits account for about 94% [5]. The global rabbit market is showing a growing trend in both volume and value, especially in Asia, North America, Europe, and other regions. Rabbit products (meat, hair, skin) have special economic value, can be exported, and play an important role in the development of economic development. Feed accounts for 60–70% of the breeding costs of meat rabbits. Corn is one of the main raw materials of feed, and its consumption is relatively large. Therefore, this experiment selected New Zealand rabbits as the research object to further reduce the breeding cost of meat rabbits while producing better quality rabbit meat.

The substitution of corn with different raw materials has different effects on the production performance, nutrient digestibility, and intestinal microbial species of animals, indicating that corn can be replaced by other raw materials without causing much hazard to animals. Replacing corn with licuri cake [6], shredded sugar beet pulp [7], elephant grass (*Pennisetum purpureum*) [8], sorghum [9], and pasta wastes [10] in the diets of lactating dairy cows, dairy calves, geese, broilers, and laying Japanese quails separately could maintain growth performance and carcass traits and positively affect intestinal health, nutrient digestibility, feed conversion, and cecal microbiota.

Corn straw is a common crop waste, which is often used in animal husbandry, and its nutritional value can be improved by pretreatment and fermentation. The digestibility and growth performance of corn stalk can be improved by CaO [11,12,13], ray, and alkaline treatments [14]. The nutritional value of corn straw can be improved by adding *Neolamarckia cadamba* leaves [15], enriching and reconstructing *epiphytic flora* [16], and mixing various fruit and vegetable wastes [17] into fermentation. Therefore, pretreatment of corn straw can promote its absorption and utilization in animals.

Our research group has developed a new set of pretreatment and fermentation processes for corn straws that can increase the content of sugar released from corn straws in vitro by 220%, which may improve the ability of corn straws to provide more energy for animals. We hypothesize that the substitution of corn with fermented corn straw in the diets of New Zealand rabbits can compensate for the energy gap between corn and fermented corn straw through enhanced nutrient digestibility, improved intestinal health, and modulation of cecal microbiota homeostasis, thereby ensuring comparable growth performance. In this study, the pretreatment and fermentation effects of fermented straw were evaluated and the proportions of different concentrations of corn straw used to replace corn were determined by assessing various physiological indicators of rabbits in each group to understand the influence of fermented corn straw as a substitute for corn in animals. The effects of fermented corn straw on animal body and intestinal metabolites and intestinal microbial diversity were also investigated through analysis of the serum metabolome, cecal content metabolome, and cecal content microbial diversity data. Our study provides a new idea for developing a novel type of energy feed that can replace corn scientifically and solve the problem of an insufficient supply of corn.

## 2. Materials and Methods

### 2.1. Animals and Experimental Design

**Figure 1:**
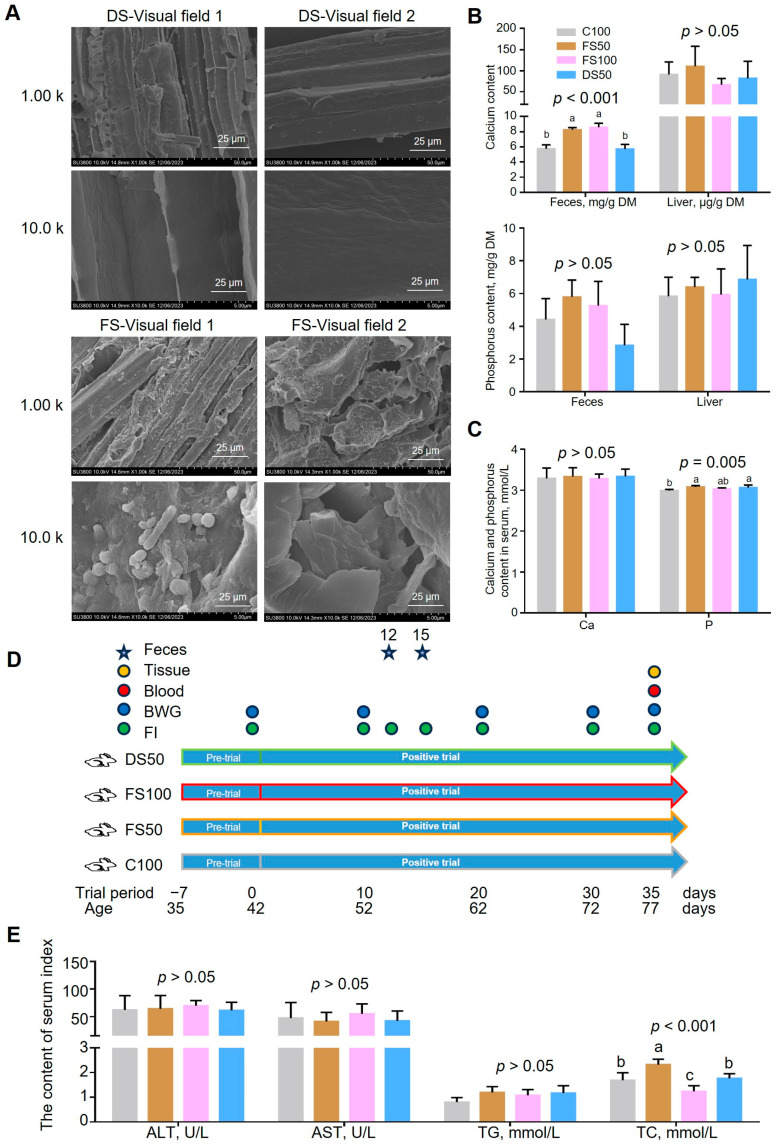
Animal experimental design and effects of excessive calcium. (**A**) Characterization and analysis of the ultrastructure of corn straw and fermented straw by scanning electron microscopy. (**B**) The content of calcium and phosphorus in the feces and liver. Significance testing was carried out using Duncan’s test (*p* < 0.05). Experiments were performed in biological triplicate, each with 6 New Zealand rabbits for each repeat. The bars represent the mean standard errors of each genotype (n = 6). (**C**) Calcium and phosphorus content in serum. (**D**) Experimental design of the effect of fermented corn straw on growth performance, digestion and absorption, and immune function of New Zealand rabbits. Colored circles and pentagrams represent samples and physiological indexes collected during the positive trial period. BWG, weight gain. FI, feed intake. (**E**) Biochemical parameters related to liver function in serum. ALT, alanine aminotransferase. AST, aspartate aminotransferase. TG, glyceryl tridodecanoate. TC, total cholesterol. Significance testing was carried out using Duncan’s test (*p* < 0.05). The letters a–c represent significant differences (*p* < 0.05).

All animal operations were performed in accordance with the experimental animal care and use guidelines of Huazhong Agricultural University, and the animal experiment protocol was approved by the Science Ethics Committee of Huazhong Agricultural University (No. HZAURAB-2024-0010, Wuhan, China). During pregnancy, 120 healthy New Zealand female rabbits were selected on the basis of their similar expected date of birth and parity. After weaning, 360 healthy 35-day-old rabbits of similar weight were selected as the starting group. They were randomly divided into 4 groups with 30 rabbits in each group and fed the corresponding diet for 7 days (prefeeding period). The experimental groups were (i) C100, (ii) FS50, (iii) FS100, and (iv) DS50, and the dietary composition of each group is shown in Table 1. During the prefeeding period, the gradient refueling method was adopted. The ratio of the experimental diet to the initial diet was 1:2 for 2 d feeding, then 2:1 for 2 d feeding, and finally, 100% experimental diet for 3 d feeding. After the prefeeding period, young rabbits with similar body weights (1135 ± 5.3 g) were selected for the experimental period, with 30 rabbits in each group, half male and half female (Figure 1D). The experimental period lasted 35 days. Rabbit houses and cages were thoroughly disinfected before the start of the experiment, and routine immunization and feeding management were conducted throughout the experiment. During the trial period, rabbits in a single metal cage (50 cm × 50 cm × 45 cm) were fed twice a day (08:00 and 18:00, respectively), with free access to drinking water, moderate feeding restrictions in the early stage, and free feeding in the later stage. Rabbit houses were naturally ventilated and well lit. The temperature was 23.10 ± 3.25 °C and the relative humidity was 63.14 ± 5.42%.

In order to reduce experimental errors and ensure reliable experimental results, randomization and blinding were used in our animal allocation and analysis. A total of 360 rabbits were randomly assigned to four experimental groups using a computer-generated sequence of random numbers. Randoms were stratified by gender to ensure that there were equal numbers of male and female rabbits in each group. The researchers were not involved in subsequent data collection or analysis, which ensured that the allocation was fair. The study was conducted as a single-blind experiment. Researchers who performed experimental procedures and collected data were aware of group assignments. However, the data analysis researchers knew about grouping only after the analysis was completed. Data statistics analysts did not know the grouping situation, which was necessary to ensure that data analysis was objective and impartial.

Corn stalks harvested in Shandong Province in 2020 were used; they were cut short after drying, mechanically crushed, and passed through a sieve to retain straw powder with a particle size of 10–40 mesh. The production process of fermented corn stalk is as follows: 100 kg of dry corn stalks and 10 kg of calcium hydroxide are added to the double-screw belt mixer, and 200 kg of tap water is added with a pump while stirring, so that the straw can fully absorb moisture and alkali evenly, and then the straw is packed into a plastic bag and sealed, and a total of 1 t of straw is treated and left to stand at 20 ± 5 °C for 7 days. The treated 310 kg straw was added to the mixer, 25 kg of puffed corn flour was evenly sprinkled while stirring, and 70 kg of bacterial solution was added to the seed solution. The seed solution contained 2 kg of *Lactobacillus plantarum* (MRS culture), 0.1 kg of yeast soaking powder, and 0.2 kg of saccharase (Angel, Yichang, Lot377691, 50,000 U/g). The mixed straw was resealed in a breathable bag and placed in solid fermentation at a temperature of 20 ± 5 °C for 15 days. *Lactipantibacillus plantarum* was preserved in the fermentation engineering room of Huazhong Agricultural University (Wuhan, China).

### 2.2. Sample Collection

At the beginning and end of the experiment, each rabbit was weighed, and its fasting weight was recorded. During the trial period, weight and feed intake were measured once a week, the diarrhea score was determined daily, and mortality was ascertained. On the 12th to 15th days of the trial period, 10 healthy rabbits, 5 male and 5 female, were selected from each group. Body weight and feed intake were recorded, fresh fecal samples were collected, 10 mL of 10% H_2_SO_4_ solution was added to every 100 g of fresh fecal sample, volatile nitrogen was fixed, and the samples were quickly frozen for the determination of nutrient digestibility. On the 35th day of the trial period, 6 rabbits from each group were weighed after fasting for 12 h and sampled after euthanasia. The rabbits were injected with phenobarbital sodium, underwent cardiac blood collection at the time when both the toe pinch and leg retraction reflexes ceased, and then exsanguinated until death. After the blood had rested on the inclined plane for 30 min, the serum was separated via centrifugation at 4 °C at 1500× *g* for 10 min and frozen at −80 °C. Samples of the duodenum, jejunum, and middle ileum approximately 1 cm in length were collected, immediately washed with precooled normal saline, fixed with 4% paraformaldehyde, and stored in a refrigerator at 4 °C for periodic acid–Schiff (PAS) staining and immunohistochemical experiments. Duodenum, jejunum, ileum mucosa, intestinal contents, cecum contents, liver, longissimus dorsi muscle samples, and liquid nitrogen quick-freezing samples were collected. The thymus, spleen, round sacculus, and vermiform appendix were collected, weighed, and recorded, and the immune organ indices were calculated. In order to reduce the impact of cage position on the rabbits, all rabbits were uniformly raised in upper cages. Sample collection employed a standardized sampling protocol, ensuring consistency and accuracy by assigning specific tasks to designated individuals, maintaining a fixed sequence of operations, and utilizing uniform tools, thereby minimizing human error.

### 2.3. Growth Performance, Nutrient Content, and Total Intestinal Apparent Digestibility

The body weights of the New Zealand rabbits were recorded on the first and final days of the trial, and the feed intake was measured every 10 days to calculate the average daily weight gain, average daily feed intake, and feed conversion ratio (FCR). The formula for the FCR is as follows: FCR = (Average daily feed intake (g))/(Average daily gain (g)).

The contents of crude fiber (CF), crude protein (CP), neutral detergent fiber (NDF), acid detergent fiber (ADF), acid detergent lignin (ADL), crude ash, acid insoluble ash (AIA), calcium (Ca), and total phosphorus (P) in the straw, diet, and feces were determined according to the analytical methods of Feed Analysis and Quality Test Technology [18]. The Ca content was determined using a flame atomic absorption analyzer, and the total P content was determined using the molybdenum yellow method [19]. The content of soluble dietary fiber in the straw was determined with reference to GB/T5009.88-2014, and the amino acid content was determined with reference to GB/T18246-2019 using an automatic amino acid analyzer. Cellulose = ADF − ADL, hemicellulose = NDF − ADF.

The apparent digestibility of the whole intestine was calculated using AIA as an endogenous indicator. The calculation formula was as follows: apparent digestibility = ((the content of a nutrient in feed/the content of AIA in feed − the content of a nutrient in feces/the content of AIA in feces)/(the content of a nutrient in feed/the content of AIA in feed)) × 100%.

### 2.4. Immune Organ Indices and Diarrhea Scores

The immune organ index was calculated as the ratio of organ weight to live weight before slaughter. The appearance and shape of the feces were observed, recorded daily, and scored according to the degree of diarrhea. The diarrhea scoring criteria are shown in Table S1. The diarrhea index was calculated as the total diarrhea score/total number of rabbits. Diarrhea incidence: (total number of rabbits with diarrhea/total number of rabbits) × 100%.

### 2.5. Determination of Serum Biochemical Indicators

On the final day of the trial, blood samples were taken and left to stand on a slant for 30 min after 12 h of overnight fasting. Then, the samples were centrifuged at 1500× *g* for 10 min at 4 °C to separate the serum for the measurement of glucose (Glu), alanine aminotransferase (ALT), aspartate aminotransferase (AST), glyceryl tridodecanoate (TG), total cholesterol (TC), total protein (TP), albumin (ALB), urea, and calcium levels using an automatic biochemical analyzer (BX-4000, Sysmex, Shanghai, China).

### 2.6. Determination of SCFA Levels

The contents of the cecum were freeze-dried by Labconco (FreeZone, Beckman, Brea, CA, USA), and the concentrations of short-chain fatty acids (SCFAs) were determined via gas chromatography (7890A, Agilent, Santa Clara, CA, USA) [20]. A J&WDB-FFAP gas chromatographic column (122-3232, Agilent, Beijing, China) was used, and the column temperature box conditions were as follows: 100 °C for 0 min, an 8 °C/min temperature increase to 150 °C for 2 min, and then 210 °C for 4 min.

### 2.7. Determination of Enzymatic Hydrolyzed Sugar in Straw Using HPLC

In total, 0.5 g of straw sample dried at 105 °C was accurately weighed into a 100 mL bottle, 50 mL of enzyme buffer was added, and the mixture was shaken at 100 r/min for 72 h. A blank group without straw was set up each time to remove the effect of the sugar brought in by cellulase. The hydrolyzed sample was diluted 5 times, and high-performance liquid chromatography (HPLC) was performed to detect the glucose and xylose content [21]. To prepare the enzyme buffer, 4 mL of β-glucanase (12311103, SUNSON, Cangzhou, China) and 4 mL of xylanase (12312037, SUNSON, Cangzhou, China) were added to 25 mL of 2 M acetic acid–sodium acetate buffer (A875409 and S817983, Macklin, Shanghai, China), and the pH of the buffer was adjusted to 4.8. Then, 0.1 g of ampicillin sodium (A800429, Macklin, Shanghai, China) was added to the above solution. The solution was mixed evenly, and distilled water was added to bring it up to 1 L. The HPLC column was a Bio-Rad HPX-87 H ion-exclusion column, and the chromatographic conditions were as follows: the mobile phase was 5 mM H_2_SO_4_, the column temperature was 40 °C, the differential refractive index detector, the detector temperature was 35 °C, the flow rate was 0.6 mL/min, and the injection volume was 20 µL.

### 2.8. Detection of Glucose, Organic Acid, pH, and Viable Bacteria in Straw Fermentation

On the 0th, 1st, 2nd, 3rd, 4th, and 5th day after inoculation, 10 g of sample was collected and placed into 250 mL shaking bottles. Then, 90 mL of sterile deionized water was added, and the mixture was shaken at 180 r/min for 10 min to extract organic acids and glucose. The extract was diluted in a gradient manner, applied to De Man, Rogosa, and Sharpe (MRS) agar culture medium, and cultured at 37 °C for 48 h, and the colonies were counted [22]. Another portion of the extract was centrifuged at 10,000× *g* at 4 °C for 2 min; the supernatant was collected, diluted 5 times, filtered using a 0.22 µm filter membrane, and analyzed using HPLC to detect the content of glucose and organic acids. The HPLC conditions were the same as those for the detection of enzymatic sugar content. The pH of the remaining extract was measured and recorded.

### 2.9. Characterization and Analysis of Ultrastructure of Corn Straw and Fermented Straw Using Scanning Electron Microscopy

Accurately weighed 10 g of straw sample dried at 105 °C was placed into a 1 L blue-capped bottle. Then, 500 mL of neutral detergent was added, mixed well, and boiled in a boiling water bath for 1 h. After cooling, the mixture was filtered through 4 layers of 150-mesh nylon mesh, washed with deionized water until there was no foam, and dried at 60 °C for 72 h. A biological scanning electron microscope (SEM) (JSM-6390LV, NTC, Tokyo, Japan) was used to characterize the structure of the straw: a single layer of the sample was laid flat on the conductive glue on the sample stage, gold was sprayed for 15 min, argon was passed, and the scanning voltage was set to 10 kV.

### 2.10. Intestinal Morphology and Immunohistochemistry

Duodenum, jejunum, and ileum samples were stored in 4% paraformaldehyde fixative for morphological analysis. The samples were trimmed, dehydrated, embedded, sliced, stained with PAS, and mounted in sequence, and two discontinuous slices were taken for each tissue. The target area of each tissue was selected using an EclipseCi-L camera microscope for 40× and 100× imaging. Using Image-Pro Plus 6.0 analysis software, a 40× field of view was selected to measure the length of 5 intestinal villi in each slice, as well as the corresponding 5 crypt depths and muscle layer thickness; a 100× field of view was selected to measure the length of 5 villus epithelia, count the corresponding goblet cells, and calculate the villus–crypt ratio and the number of goblet cells per unit length.

The immunohistochemical experimental steps for thejejunum and ileum tissues were as follows: paraffin sections were placed in a repair box containing 0.01 mol/L citric acid solution (pH 6.0) in a microwave oven for antigen repair. The sections were blocked with 3% BSA diluted in PBS at room temperature for 1 h, then incubated with the primary antibody against MUC2 (1:1500, GB11344, servicebio, Wuhan, China) or ZO1 (1:2000, 21773-1-AP, Sanying, Wuhan, China) at 4 °C overnight, washed, and incubated with the corresponding secondary antibody at room temperature for 50 min. The sections were then developed with DAB, counterstained with cell nuclei, dehydrated, mounted, and finally placed under a white light microscope for scanning (GDP2201, Servicebio, Wuhan, China). A Case Viewer was used to scale and crop images, and Image-Pro Plus 6.0 was used to measure and calculate the average optical density (AOD). AOD = integrated optical density (IOD) sum/area sum.

### 2.11. Gene Expression Analysis by Real-Time PCR

Total RNA was extracted from jejunum and ileum mucosa samples (100 mg) using the RNAeasy™ animal RNA extraction kit (R0027, Beyotime, Shanghai, China). The concentration and quality of total RNA in the jejunum and ileum mucosa were determined using a microspectrophotometer (Nnano-1000, UMI Instrument, Hangzhou, China). The HiScript III 1st Strand cDNA Synthesis Kit was used to reverse transcribe total RNA (1 µg) into cDNA (R312-01, Vazyme, Nanjing, China). The 2× Universal SYBR Green Fast qPCR Mix (RK21203, ABclonal, Wuhan, China) was used for real-time quantitative PCR (RT-qPCR) analysis. The primers used in the study are shown in Table S2. GAPDH was used as the internal reference and the 2^−ΔΔCt^ value was used to calculate the target gene expression [23,24].

### 2.12. Determination of Microbial Protein Content and Microbial Enzyme Activity

Cecal contents were processed for microbial protein extraction and enzyme activity assays. For protein extraction, a 0.1 g freeze-dried cecal sample was suspended in 1 mL sterile PBS (pH 7), centrifuged at 408× *g* for 5 min at 4 °C, and the supernatant was further centrifuged at 25,000× *g* for 40 min at 4 °C. The pellet was resuspended in 1.5 mL 0.25 M NaOH, boiled for 10 min to lyse cells, and centrifuged at 25,000× *g* for 60 min at 4 °C. The supernatant was analyzed for protein content using a BCA kit (PA115, TIANGEN, Beijing, China).

Enzyme activities (CMCase, MCCase, xylanase, pectinase) were measured using the DNS method [25,26]. Briefly, a 0.5 g freeze-dried cecal sample was suspended in 5 mL PBS (pH 7), sonicated on ice at 30% intensity for 5 min (3 s on, 5 s off) using an Ultrasonic Cell Crusher (SCIENTZ-IID, SCIENTZ, Ningbo, China), and centrifuged at 12,000× *g* for 10 min at 4 °C. The supernatant was used as a crude enzyme solution. For each enzyme, 0.2 mL substrate (sodium carboxymethyl cellulose, microcrystalline cellulose, xylan, or pectin), 0.6 mL PBS (pH 7), and 0.2 mL crude enzyme solution were mixed and incubated at 39 °C for 30 min. After adding 0.4 mL DNS solution and boiling for 5 min, absorbance was measured at 540 nm. Enzyme activities were calculated based on reducing sugar release using standard curves of D-glucose, D-xylose, and D-galacturonic acid.

### 2.13. DNA Extraction and 16S rRNA Sequencing

Genomic DNA extraction of cecum contents, DNA purity, PCR amplification, library preparation, and sequencing analysis were commissioned to Metware (Wuhan, China). Among these, DNA extraction used the genomic DNA extraction kit (DP336), DNA purification used the Universal DNA purification and recycling kit (DP214-03), and PCR amplification used the Phusion^®^ High-Fidelity PCR Master Mix (M0532S, New England Biolabs, Inc., 240 County Road, Ipswich, Australia). Genomic DNA was used as a template, and PCR amplification was performed using forward primer 515F (5′-GTGYCAGCMGCCGCGGTAA-3′) and reverse primer 806R (5′-TCGGACTACHVGGGTWTCTAAT-3′) to amplify the V4 region of the 16S rRNA gene by specific primers. The research used the NEBNext Ultra II DNA Library Prep Kit (No. E7645B) to prepare libraries. After qualifying, NovaSeq6000 was used for on-machine sequencing. Cecum content microbial diversity sequencing data were stored in the National Center for Biotechnology Information (NCBI) (PRJNA1177971), accessed on 24 December 2026, (https://dataview.ncbi.nlm.nih.gov/object/PRJNA1177971?reviewer=lne59unqhu05fuaisie03hqerm).

### 2.14. Metabolite Extraction and Analysis

Serum non-targeted metabolome was completed by Majorbio (Shanghai, China), and cecal content metabolome was completed by Metware (Wuhan, China). The data reported in this paper have been deposited in OMIX, China National Center for Bioinformation/Beijing Institute of Genomics, Chinese Academy of Sciences(https://ngdc.cncb.ac.cn/omix. Accession no. OMIX009633, accessed on 10 March 2027. Accession no. OMIX009634, accessed on 19 May 2027.)

### 2.15. Statistical Analysis

The data in the table are presented as means ± SDs. All data, including cecal microflora diversity data and metabolite data, were analyzed using ANOVA with the general linear model program in SPSS 22.0 (IBM Inc., Armonk, New York, NY, USA), and the significance level was set at 0.05. For the analysis of the serum metabolome data, all metabolites were analyzed using multigroup differential analysis without data conversion or one-way analysis of variance (one-way ANOVA). Scheffe statistical software was used for the post hoc test.

## 3. Results

### 3.1. Pretreatment and Fermentation Improved the Nutritional Value of Corn Stalks

The sugar concentration from the enzymolysis of pretreated straw increased by 220% (*p* < 0.001) (Figure S1A). After inoculation, the gradual release of glucose from cornmeal provided energy for the proliferation of Lactobacillus plantarum. As the bacterial population expanded and consumed glucose, the glucose concentration in the system gradually decreased to 11.8 mg/g dry straw (DS). The viable cell count peaked at 3.22 × 10^9^ CFU/g wet material within the first 2 days and subsequently declined to 8.82 × 10^8^ CFU/g wet material by day 5 of fermentation. These results indicate that Lactobacillus plantarum was effectively cultured in the fermentation system, achieving the desired fermentation outcomes. The concentration of lactic acid in the fermentation system reached as high as 139 mg/g DS, accompanied by the production of 11.8 mg/g DS acetic acid. Throughout the fermentation process, the pH of the system shifted from an initial alkaline state (approximately 10.4) to an acidic state, eventually stabilizing at around 5.0 after 5 days of fermentation (Figure S1B). The generation of organic acids not only decreased the pH but also facilitated feed preservation, enhanced palatability, and provided energy for animal growth. The fibrous structure of fermented corn straw exhibited more pores and a rougher surface (Figure 1A). Compared with dry straw (DS), the fiber content of fermented straw was significantly reduced by 8.05% (*p* < 0.05), indicating that the fibrous components of the fermented straw were effectively degraded (Table S3). In addition, to facilitate the application of fermented straw in the animal breeding industry, the composition of 17 hydrolyzed amino acids was determined using high-performance liquid chromatography, and the results are shown in Table S4.

### 3.2. Dietary Composition, Growth Performance, and Digestibility

The dietary CF, NDF, and ADF contents increased in the FS50 and FS100 groups, and the crude protein content (mean 15.55% DM) was similar among the four experimental groups (Table 1). After 35 days of the experiment, the final weight, average daily gain, and average daily feed intake in the FS100 group were significantly greater than those in the other groups, and the feed coefficient also showed a downward trend, while there was no significant difference (the actual *p* values ranged from 0.451 to 0.949) in the above indices among the C100, FS50, and DS50 groups (Table 2). Fermented straw increased the total intestinal apparent digestibility of CP but decreased the total intestinal apparent digestibility of DM, CF, NDF, and ADF and had no significant effect on the mRNA expression levels of genes related to glucose metabolism and lipid metabolism in meat (Figure S2). These results indicate that the complete replacement of corn with fermented corn straw in the diet of New Zealand rabbits is conducive to enhancing growth performance.

### 3.3. Effects of Excess Calcium in Fermented Straw

The calcium content in fermented straw was 5.8 times greater than that in dry straw (Table S3). To determine whether excess calcium could have adverse effects on animals, we measured the calcium and phosphorus levels in the feces, liver, and serum. FS100 significantly increased fecal calcium content (*p* < 0.001) by 49.4% compared to the C100 group, but had no significant effect on the liver or serum calcium content (Figure 1B,C). Moreover, there were no significant differences in the serum biochemical indices, such as ALT (*p* = 0.910), AST (*p* = 0.838), TG (*p* = 0.218), TP (*p* = 0.628), ALB (*p* = 0.770), UREA (*p* = 0.068), or glucose (*p* = 0.058), among the groups (Figure 1E and Figure S3A). This finding indicates that excess calcium can be excreted by animals through feces, thereby avoiding the adverse effects caused by retention in the body. There was no significant difference in the phosphorus content in the feces (*p* = 0.092) or liver (*p* = 0.600) between the groups, but the serum phosphorus content in the three groups supplemented with straw (FS50, FS100, and DS50) was significantly greater than that in C100 (Figure 1B,C), and the highest group improved by 3%.

### 3.4. Effect of Fermented Straw on the Micromorphology of the Jejunum and Ileum

**Figure 2:**
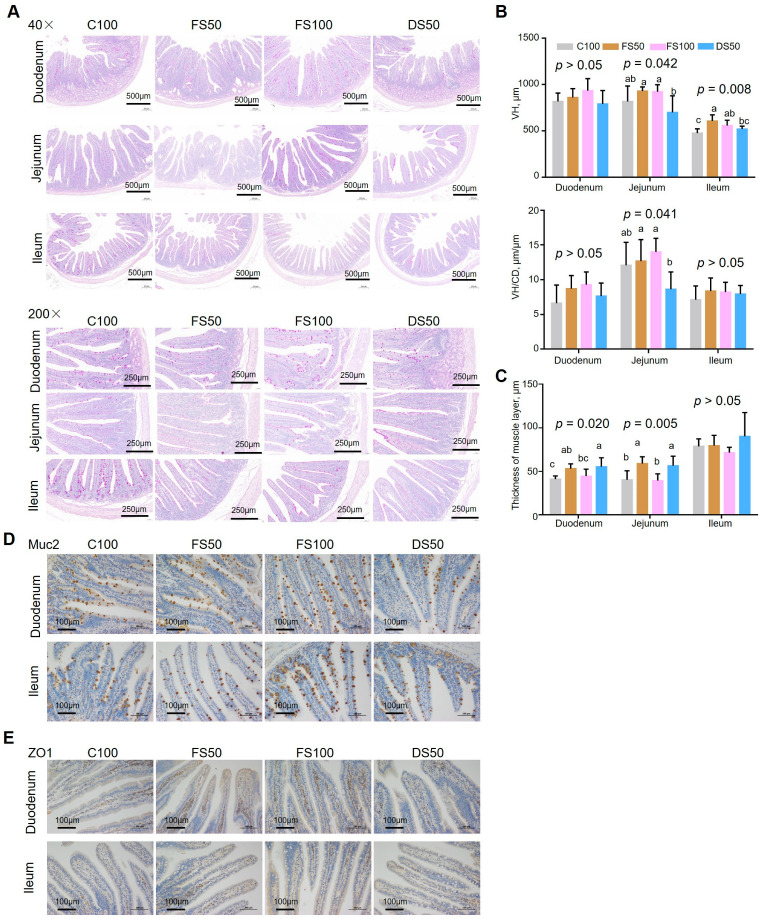
Effect of fermented straw on the morphological structure and intestinal mucosal barrier of the small intestine in New Zealand rabbits. (**A**) PAS staining of the duodenum, jejunum, and ileum. (**B**) Villus height and VH/CD of the duodenum, jejunum, and ileum. VH, villus height. CD, crypt depth. The bars represent the mean standard errors of each group (n = 6). (**C**) Thickness of the muscle layer of the duodenum, jejunum, and ileum. (**D**) Immunohistochemical detection of Muc2 in jejunum and ileum sections. (**E**) Immunohistochemical detection of ZO1 in jejunum and ileum sections. The letters a–c represent significant differences (*p* < 0.05).

Fermented straw increased the villus height of the jejunum and ileum and the VH/CD ratio of the jejunum (Figure 2A,B) but had no significant effect on the crypt depth of the duodenum, jejunum, ileum, or thickness of the muscle layer (Figure 2C and Figure S3B,C). FS100 increased the villus height by 32.1% and the VH/CD ratio by 27.3% in the jejunum compared to the DS50 group, while FS50 increased the villus height of the ileum by 27.3% compared to the C100 group. These results indicate that the increase in fiber components in the diet and the increase in intestinal chyme-empting speed reduced the concentration of nutrients in the intestine. After the body fell, the surface area of the intestinal villi increased with increasing height and VH/CD ratio of the intestinal villi, and the nutrient absorption rate improved.

### 3.5. Effects of Fermented Straw on VFAs and Microbial Enzyme Activities in the Cecum

**Figure 3:**
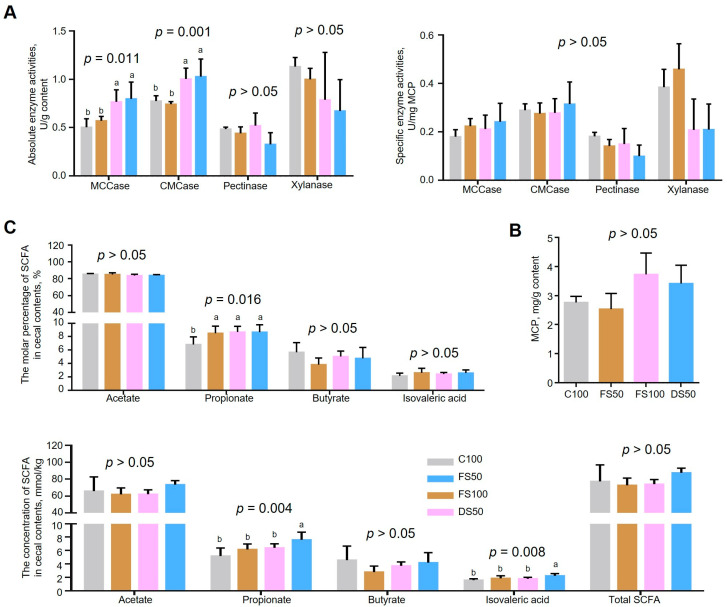
Cecal fermentation parameters and microbial enzyme activity of cecal contents. (**A**) Microbial enzyme activity of cecal contents. MCCase, microcrystalline cellulase. CMCase, carboxymethyl cellulase. (**B**) The content of microbial protein in cecal contents. MCP, microbial protein. (**C**) The content and molar percentage of SCFA in cecal contents. The bars represent the mean standard errors of each group (n = 6). The letters a–c represent significant differences (*p* < 0.05).

Fermented straw significantly increased the molar percentage of propionic acid in the cecum contents (*p* = 0.016), but had no significant effect on the molar percentages of acetic acid, butyric acid, or isobutyric acid (*p* > 0.05) (Figure 3C). FS100 increased the molar percentage of propionic acid in the cecum contents by 27% compared to the C100 group. Fermented straw had no significant effect on the content of microbial protein in the cecum contents (*p* = 0.088) (Figure 3B) and had no significant effect on the activities of pectinase and xylanase. But FS100 increased the activities of microcrystalline cellulase by 51% and carboxymethyl cellulase by 28% compared to the C100 group (*p* < 0.05) (Figure 3A).

### 3.6. Effects of Fermented Straw on the mRNA Expression Levels of Nutrient Transport-Related Genes

To further understand the effects of fermented straw on nutrient digestion and absorption, the mRNA expression levels of genes related to glucose transporters, amino acid transporters, fatty acid transporters, and the mitochondrial electron transport chain in the jejunum and ileal mucosa were measured (Table 1). FS100 decreased the mRNA expression levels of the glucose transporter-related gene solute carrier family 2 member 2 (*SLC2A2*) by 21.6% (*p* = 0.009) in the jejunum mucosa, solute carrier family 7 member 5 (*SLC7A5*) by 76.9% (*p* = 0.006) in the ileal mucosa, and microtubule-associated scaffold protein 2 (*MTUS2*) by 62.5% (*p* = 0.018) in the ileal mucosa compared to the C100 group. FS100 decreased the mRNA expression levels of the mitochondrial electron transport chain-related gene NADH:ubiquinone oxidoreductase subunit A13 (*NDUFA13*) by 46.7% (*p* = 0.041) in the jejunum mucosa compared to the C100 group. FS100 decreased the mRNA expression levels of the mitochondrial electron transport chain-related genes NADH dehydrogenase 1 alpha subcomplex subunit 1-like (*LOC108177067*) by 95.8% (*p* = 0.001), NADH:ubiquinone oxidoreductase subunit A6 (*NDUFA6*) by 33.3% (*p* = 0.001), NADH:ubiquinone oxi-doreductase subunit A13 (*NDUFA13*) by 52% (*p* < 0.001) and NADH dehydrogenase [ubiquinone] 1 beta subcomplex subunit 1 (*LOC103348994*) by 37.5% (*p* < 0.001) in the jejunum mucosa compared to the C100 group (Figure S4). These results indicate that the increase in fiber components in the diet led to an increase in the emptying speed of chyme, and there were not enough nutrients to be transported, so the expression of nutrient transporters decreased, and the expression of the mitochondrial electron transport chain, which provides energy for them, also decreased.

### 3.7. Effect of Fermented Straw on Immune Function

**Figure 4:**
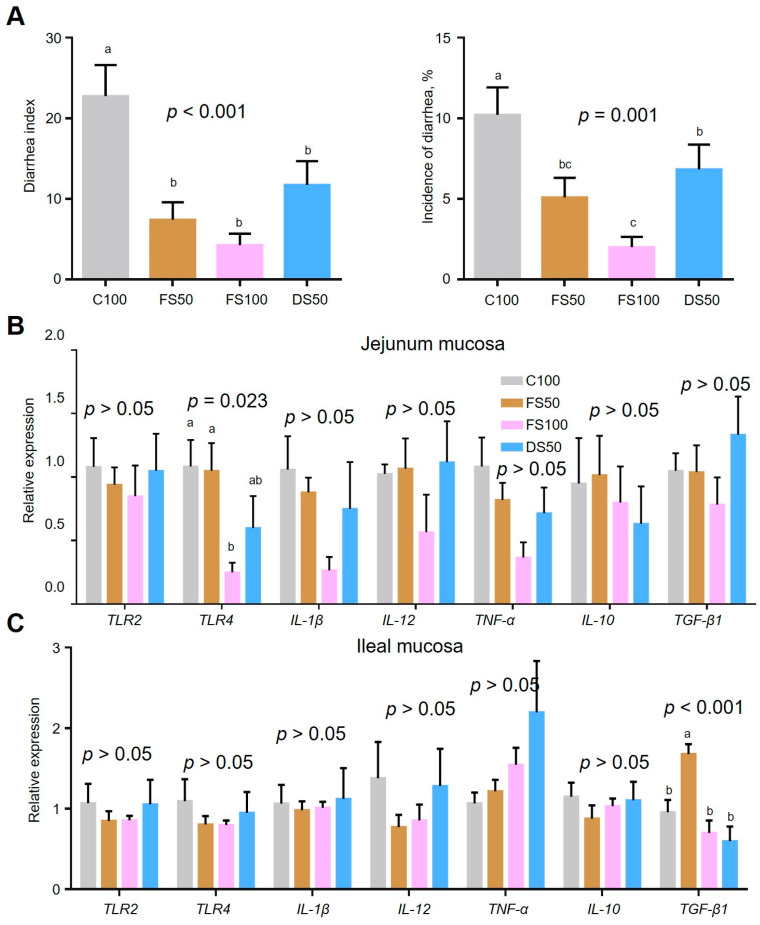
Effect of fermented straw on the immune function of New Zealand rabbits. (**A**) Effects of fermented straw on the diarrhea index and incidence of diarrhea in New Zealand Rabbits. (**B**) Detection of inflammation-related gene expression in the jejunal mucosa. (**C**) Detection of inflammation-related gene expression in the ileal mucosa. The letters a–c represent significant differences (*p* < 0.05).

Fermented straw reduced the diarrhea index and diarrhea incidence in weaned offspring rabbits (Figure 4A), and FS100 decreased the diarrhea index by 81.1% (*p* < 0.001) and diarrhea incidence by 80.4% (*p* = 0.001) in weaned offspring rabbits compared to the C100 group. FS100 also decreased the spleen indices by 35.6% (*p* = 0.002) compared to the C100 group, whereas the FS50 and FS100 groups showed no change, indicating that pretreatment with and fermentation of straw could alleviate the adverse effects of straw on the immune function of New Zealand rabbits (Table S5). There were no significant differences in the thymus index (*p* = 0.226), vermis process index (*p* = 0.673), or round sac index (*p* = 0.188). FS100 decreased the mRNA expression levels of *TLR4* by 76.9% (*p* = 0.023) in the jejunum mucosa compared to the C100 group, and FS50 increased transforming growth factor beta-1 (TGF-β1) by 75.7% (*p* < 0.001) in the ileal mucosa compared to the C100 group. The mRNA levels of toll-like receptor 2 (*TLR2*), interleukin 1 beta (*IL-1β*), interleukin 12 (*IL-12*), tumor necrosis factor (*TNF*), and interleukin-10 (*IL-10*) were not significantly different among the groups. These results indicate that fermented straw could promote the immune function of animals to some extent (Figure 4B,C).

### 3.8. Effects of Fermented Straw on the mRNA Expression and Protein Expression of Intestinal Barrier-Related Genes

FS100 increased the AOD of MUC2 expression in the ileum by 25.5% (*p* = 0.037) compared to the DS50 group (Figure S3D), whereas the AOD of MUC2 in the duodenum and the ZO1 tight junction protein in the duodenum and ileum did not significantly differ among the groups (Figure 2D,E, Figures S3D and S10E). Moreover, the mRNA expression levels of genes related to the intestinal physical barrier in the jejunum and ileum mucosa did not differ among the groups (Figure S5). These results indicate that fermented straw could enhance intestinal mucosal immunity by enhancing the chemical barrier of the ileum.

### 3.9. Effects of Fermented Corn Stalk on Serum Metabolomics

**Figure 5:**
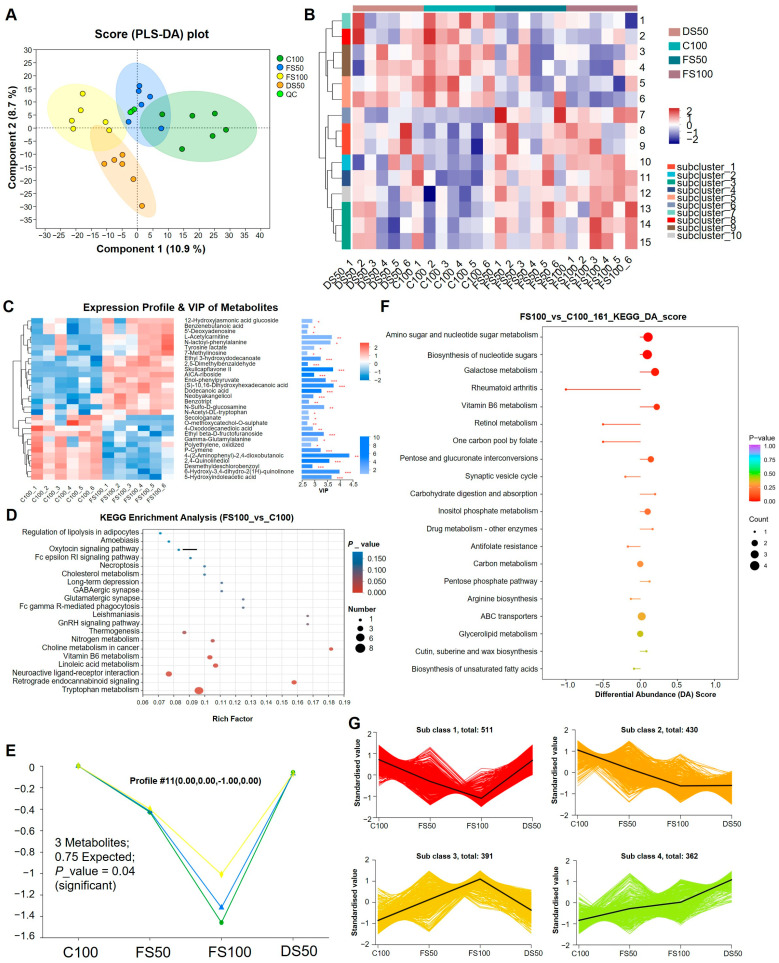
Metabolome analysis of serum and cecal contents. (**A**) PLS-DA score chart. (**B**) Metabolite cluster analysis diagram. 1: CMP-3-deoxy-D-manno-octulosonate, 2: UK-121,265, 3: 5-Hydroxyindoleacetic acid, 4: Methyl bisnorbiotinyl ketone, 5: Bimakalim, 6: Desmethyldeschlorobenzoyl indomethacin, 7: Contignasterol, 8: Risbitin, 9: LysoPC (16:1 (9Z)/0:0), 10: Dihydrozeatin, 11: Tributyl citrate, 12: Wedelolactone, 13: Pyroglutamyl-prolyl-arginine-4-nitroanilide, 14: 2-Aminoacetophenone, 15: 6-Fluorohomovanillic acid. (**C**) VIP value analysis diagram of C100 and FS100 differential metabolites. On the right, * represents *p* < 0.05, ** represents *p* < 0.01, and *** represents *p* < 0.001. (**D**) KEGG pathway topology analysis bubble diagram of the metabolic set FS100_vs_C100. Each bubble in the figure represents a KEGG pathway. (**E**) Profile 11 trend chart. Green, blue, and yellow lines represent different metabolites, respectively. (**F**) KEGG cluster of unique differential metabolites of fermented straw. The p-value is the hypergeometric test p-value, and the top 20 pathways ranked by p-value are displayed from smallest to largest. (**G**) K-means map of differential metabolites. The graphs show the standardized value changes of four subclasses (Sub class 1, 2, 3, 4) under different enrichment factors (C100, FS50, FS100, DS50). Each subclass is represented by a different color: red (Sub class 1), orange (Sub class 2), yellow (Sub class 3), and green (Sub class 4). The black line indicates the average trend for each subclass.

The degree of separation of the samples in each group was relatively large (Figure 5A), indicating that the addition of straw had a greater effect on the metabolic profile of New Zealand rabbits. FS100 with C100 increased the number of differential metabolites by 146% compared to the FS50 group, indicating that the number of host serum metabolites increases as more fermented straw was added (Figure S6A). The number of differential metabolites caused by the straw itself was 20 (Figure S6C), and the only difference in metabolites between the fermented straw group and the C100 group was 30 (Figure S5D). A cluster analysis was performed on the 30 differential metabolites unique to the fermented straw group. Among the upregulated differential metabolites in the FS100 group, the significantly different metabolites were 6-fluorohomovanillic acid, lyso PC (16:1 (9z)/0:0), wedelolactone (Figure 5B), skullcapflavoni (SFII), and (s)-10,16-dihydroxyhexadecanoic acid (Figure 5C). Among the downregulated differential metabolites in the FS100 group, the significantly differential metabolites were 4-(2-aminophenyl)-2,4-dioxobutanoic acid and 6-hydroxy-3,4-dihydro-2(1H)-quinolinone (Figure 5C).

BH multiple testing correction of the metabolic set FS100_vs_C100 revealed significant enrichment of metabolic pathways related to tryptophan metabolism, retrograde endocannabinoid signaling, and neural activity of ligand–receptor interaction pathways (Figure 5D and Figure S5D). We analyzed the metabolic set FS100_VS_C100 in the pretreated data table, which contained 260 differential metabolites. By analyzing the temporal expression trends of these metabolites, we found that the temporal pattern of cluster number 11 was consistent with the significant change trend (Figure S6E). Figure 5E shows three significantly downregulated metabolites in fermented straw. They were 4-(2-aminophenyl)-2,4-dioxobutanoic acid, 6-hydroxy-3,4-dihydro-2(1H)-quinolinone, and 2,4-quinolinediol.

### 3.10. Effects of Fermented Corn Stalks on the Metabolomics of Cecal Contents

Table S6 shows information on the number of metabolites detected. The trend of metabolite aggregation between the groups was separate, and the differences in metabolites within the sample group were smaller than those between the sample groups (Figure S6F). Compared with the C100 group, the FS50, FS100, and DS50 groups presented greater separability (Figure S6G–I). The intersection of the FS50, FS100, and C100 groups included 260 different metabolites, and after 99 different metabolites between DS50 and C100 were deducted, the number of specific metabolites associated with fermented straw was 161 (Figure S6J). The genes enriched with more than 161 metabolites showed the most significant differences in the following KEGG pathways: amino sugar and nucleotide sugar metabolism, nucleotide sugar biosynthesis, and galactose metabolism (Figure 5F).

To study the trend of the changes in the relative contents of metabolites across different samples, the relative contents of all differential metabolites screened by *VIP* > 1 and *p* < 0.05 in all comparison groups were standardized using unit variance scaling (UV). K-means clustering analysis was then performed (Figure 5G). The relative contents of 511 metabolites in subclass 1 were similar in the C100 and DS50 groups but downregulated in FS50 and FS100. The relative amounts of 391 metabolites in subclass 3 were similar in the C100 and DS50 groups but upregulated in FS50 and FS100. KEGG pathway enrichment analysis of these metabolites revealed that the metabolites whose expression was downregulated in FS50 and FS100 were enriched mainly in the tryptophan metabolism protein digestion and absorption, caffeine metabolism, and aminoacyl-tRNA biosynthesis pathways (Figure S6K). The metabolites upregulated in FS50 and FS100 were enriched mainly in galactose metabolism, the biosynthesis of nucleotide sugars, amino sugar and nucleotide sugar metabolism, and vitamin B6 metabolism (Figure S6L).

### 3.11. Effects of Fermented Corn Stalks on Microbial Diversity in the Cecum

**Figure 6:**
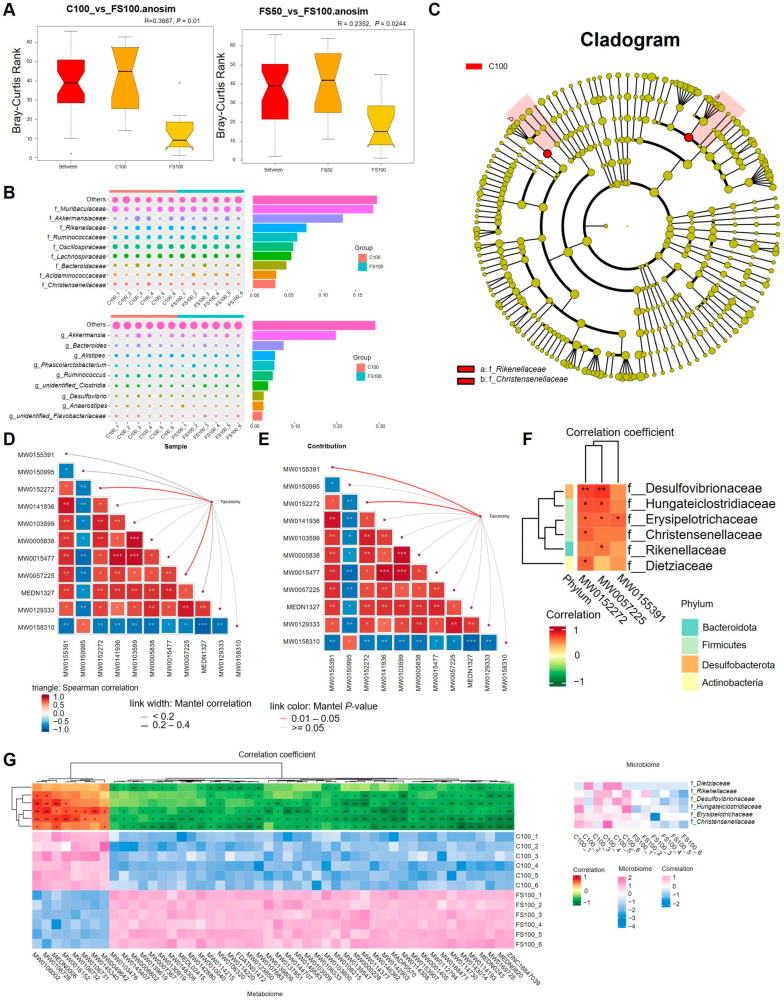
Microbiome analysis of cecum contents and combined analysis of microbiome and metabolome. (**A**) Significance test of Anosim community structure differences between groups. The “+” symbol indicates outliers, which are values that deviate significantly from other data points. (**B**) Analysis of species contribution to differences. The results showed the top 10 species with the highest contribution to the difference between the two groups and their abundance. (**C**) Evolutionary cladistics of LEfSe (LDA effect size) for intergroup differential species. (**D**,**E**) Mantel test analysis of taxa and single metabolites of different microorganisms in FS100_vs_C100. (**F**) Spearman correlation clustering heat maps of 3 different metabolites and FS100_vs_C100 differential microorganisms showed the magnitude of the Spearman correlation between different microorganisms and different metabolites. (**G**) Spearman correlation clustering heat maps of FS100_vs_C100 differential microorganisms and differential metabolites. * represents *p* < 0.05, ** represents 0.001 < *p* < 0.01, *** represents *p* < 0.001.

The difference in the cecal microbial community structure between the C100_vs_FS100 and FS50_vs_FS100 groups was significantly greater than the difference within the groups (Figure 6A), whereas the difference between the other groups was not significant (Figure S7C), indicating that compared with the control group, the addition of fermented straw significantly changed the cecal microbial community structure (*p* = 0.01). T-test analysis of different species between groups revealed that *Desulfovibrionia*, *Desulfovibrionales,* and *Desulsipelotrichales* significantly decreased the amount of *Desulfobacterota* in the cecal contents. The abundance of f_*Desulfovibrionaceae*, f_*Erysipelotrichaceae*, and g_*unidentified_Lachnospiraceae* was unclassified (Figure S8A). Fermented straw significantly reduced the abundance of harmful bacteria (*Desulfobacterota*, *Erysipelotrichales*, and *Hungateiclostridiaceae*) in the cecum. The C100 and FS100 groups differed in terms of f_*Muribaculaceae* and g_*Akkermansia* (Figure 6B). Analysis of the LDA effect size (LEfSe) results revealed that the biomarkers with significant differences between the C100 and FS100 groups were f_*Rikenellaceae* and f_*Christensenellaceae* (Figure 6C and Figure S7B,C). The abundance of both bacteria decreased significantly in the FS100 group, and their abundance was greater in the intestines of patients with arthritis and depression. Fermented straw reduces the abundance of harmful bacteria associated with arthritis in the cecum and may be helpful in preventing joint diseases in animals.

Metastats analysis of intergroup differential species revealed that the relative abundances of p_*Bacteroidota*, c_*Bacteroidia,* and s_*Coprobacter_secundus* in the FS100 group were significantly greater than those in the C100 group (Figure S8D). Additionally, *Desulfobacterota*, c_*Desulfovibrionia*, o_*Desulfovibrionales*, o_*Erysipelotrichales*, and o_*Corynebacteriales* were identified in the FS100 group, including f_*Desulfovibrionaceae*, f_*Erysipelotrichaceae*, f_*Rikenellaceae*, and g_*unidentified_Lachnospiraceae*. The relative abundances of s_*Alistipes_indistinctus*, s_*bacterium_YE57,* and s_*Clostridiales_bacterium_CIEAF_020* were significantly lower than those in the C100 group (Figure S9). Fermented straw promotes the growth of beneficial microorganisms such as *Coprobacter* in the cecum and reduces the abundance of harmful microorganisms such as *Erysipelotrichia* and *Corynebacteriales*, which is conducive to balancing the homeostasis of cecum microorganisms and reducing the occurrence of diseases.

Functional annotations and samples of Tax4Fun2 (v1.1.5) based on amplicon sequence variants (ASVs) were clustered, and the functional clustering results revealed that the functions of C100 and DS50 were similar and that the functions of FS50 and FS100 were also similar, indicating that fermented straw changed the functions of cecal microorganisms. At level 1, fermented straw increased butanoate metabolism, with butyric acid, valine, leucine, and isoleucine degradation, the abundance of intestinal microbes related to leucine and isoleucine degradation, and fatty acid metabolism. The abundance of gut microbes associated with the biosynthesis of antibiotics, purine metabolism, and metabolic pathways of antibiotics was reduced (Figure S7A). At level k, fermented straw increased the abundance of gut microbes associated with K21572, K21573, K00059, and K07636 and decreased the abundance of gut microbes associated with K02013 (Figure S7B).

### 3.12. Multiomics Analysis of the Serum Metabolome, Cecal Content Metabolome, and Cecal Content Microbiome

Differences in cecal contents between the FS100 and C100 groups were determined using Mantel test analysis of single metabolites of microbes and cecal contents, which revealed that differential microbes associated with MW0152272 (Leu-Asp-Val-Lys-His), MW0057225 (1,2-Dieicosenoyl-SN-glycero-3-phosphocholine), and MW0155391 (Phe-Tyr-Phe-Lys-Ile) were significantly associated with each other (Figure 6D,E). f_*Desulfovibrionaceae*, f_*Hungateiclostridiaceae*, f_*Erysipelotrichaceae*, f_*Christensenellaceae*, f_*Rikenellaceae,* and f_*Dietziaceae* affected the concentrations of the above three metabolites, thereby altering the cecal metabolic environment (Figure 6F). The FS100_vs_C100 differential metabolites were significantly correlated with f_*Dietziaceae*, f_*Rikenellaceae*, f_*Desulfovibrionaceae*, f_*Hungateiclostridiaceae*, f_*Erysipelotrichaceae*, f_*Christensenellaceae*, g_*Roseburia,* and g_*Sporobacter* (Figure 6G and Figure S7E,F). Detailed information corresponding to the metabolite number is shown in Tables S7–S9.

The metabolome data and microbial diversity data of the cecal contents were compared with the serum metabolome data for Procrustes analysis to determine whether the microbial diversity and metabolites of the cecal contents affected the serum metabolites of New Zealand rabbits. The lines between the serum metabolome data, cecal content metabolome data, and 16S diversity data were long, indicating low consistency among the datasets (Figure S10A,B). There was no significant change trend between the serum metabolome and cecal content metabolome and ASV diversity, and the correlation between the serum metabolome and cecal content metabolome and ASV diversity was very low. These data suggest that there is no consistency between the serum metabolome and cecal content metabolome and ASV diversity.

## 4. Discussion

### 4.1. Effects of Fermented Corn Straw on Nutrient Digestion and Absorption

Alkali treatment with water disrupted the fibrous structure of the straw, enhancing the accessibility of cellulose and hemicellulose. Concurrently, alkali-tolerant cellulose-degrading bacteria inherent in the straw surface proliferated, breaking down a portion of the cellulose to produce fermentable sugars. These sugars were subsequently utilized by the inoculated Lactobacillus plantarum to conduct lactic acid fermentation, which lowered the pH and inhibited the growth of harmful microorganisms. As the pH decreased, the majority of cellulose-degrading bacteria, which thrive in neutral to slightly alkaline environments, continued to break down cellulose, generating more fermentable sugars.

The digestibility of the fiber components in the fermented straw group decreased, which may have been directly caused by the increase in the content of these components in the formula. Increasing the fiber content of the diet can increase its presence in the intestinal cavity, speed up the emptying of intestinal contents, and shorten the residence time of chyme in the intestine [27].

Our study shows that excess calcium can be excreted by animals in their feces, thereby preventing the adverse effects of calcium retention in the body. There was no significant difference in the phosphorus content in the feces and liver between the groups, but the serum phosphorus content in the three groups supplemented with straw (FS50, FS100, and DS50) was significantly greater than that in the C100 group, which may be due to the shortened residence time of chyme in the intestine after straw replaced corn, resulting in a slower absorption rate of phosphorus in chyme [28].

The total apparent intestinal digestibility of NDF was lower in the FS100 group than in the C100 group, which may be due to the increase in the dietary fiber component. However, the increase in cellulase activity in the cecum promoted the digestion and absorption of NDF in the posterior intestine, increasing the digestibility of NDF by 15.6%. The overall growth performance of the animals manifested as an increase in feed intake and body weight and a decrease in the ratio of feed to gain. Moreover, after the fibers are degraded in the posterior intestine, more propionic acid is produced. The metabolome data of the cecal contents revealed that fermented straw increased the metabolism of carbohydrates (galactose, amino sugar, and nucleotide sugar) in the cecum. Vitamin B6 metabolism and nucleotide sugar biosynthesis decreased protein digestion and absorption, amino acid metabolism (mainly tryptophan metabolism), caffeine metabolism, and protein translation, suggesting that fermented straw reduced protein synthesis, digestion, and digestion in the cecum. Functions related to absorption and amino acid metabolism increase the number of physiological processes, such as carbohydrate metabolism and gene synthesis.

### 4.2. Fermented Straw Promotes the Production of a Variety of Beneficial Metabolites and Reduces the Concentration of Harmful Metabolites in the Body

The increased content of 6-fluorohomovanillic acid in the serum of the fermented straw group may help animals maintain a better mental state and, at the same time, maintain intestinal homeostasis and reduce inflammation and oxidative stress, which has a positive impact on animal health. Its unique fragrance may promote the animals’ appetites [29,30,31]. 2-Aminoacetophenone has a sweet and grape flavor [32] as well as antibacterial and anticancer properties [33]. Lyso PC (16:1 (9Z)/0:0) plays a role in lipid signaling by acting on the lysophospholipid receptor (LPL-R), which promotes intracellular calcium mobilization and increases insulin secretion and has anti-inflammatory effects [34,35]. Wedelolactone has antihepatotoxic, antihypertensive, antitumor, antiphospholipase A2, and antivenin properties [36,37,38]. SFII has anti-inflammatory effects, reduces pathological DNA damage and cell apoptosis, and regulates osteoclast formation, survival, and absorption [39,40,41]. Fermented straw is digested by the animal body and intestinal microorganisms, may produce scents such as mint or flavors such as sweet grape, and may improve the animals’ appetites. By participating in the regulation of reactive oxygen species and integrin signaling and other mechanisms, it produces anti-inflammatory, antibacterial, anti-tumor, hormone-regulating, calcium-mobilizing, and other bioactive metabolites, maintains microbiota homeostasis, reduces the production of harmful metabolites, prevents potential visceral diseases, protects the intestinal barrier, and maintains the health of the animal body.

4-(2-Aminophenyl)-2,4-dioxobutanoic acid is involved in tryptophan metabolism, resulting in impaired intestinal barrier function, disruption of microbiota homeostasis, and inflammation [42]. 2,4-Quinolinediol, a precursor of virulence factors that can be produced in both aerobic and anaerobic environments, is highly expressed in the sputum samples of cystic fibrosis patients with a history of positive *Pseudomonas aeruginosa* cultures [43] and in the supernatant of multiple strains of *Pseudomonas aeruginosa* and *Staphylococcus aureus* monocultures in synthetic cystic fibrosis medium (SCFM) [44].

6-Hydroxy-3,4-dihydro-2(1H)-quinolinone is a quinoline compound, and we boldly speculate that this compound and its derivatives are substances that are not conducive to body health. The levels of the above three substances in the plasma of the FS100 group decreased, indicating that fermented straw improved intestinal barrier function and microflora homeostasis, improved immune regulatory function, and reduced the inflammatory response in New Zealand rabbits. However, since this study reached the above conclusions through multiomic analysis, more direct evidence is needed to explain the effect of fermented straw on the metabolic network in animals.

### 4.3. Fermented Straw Promoted Animal Health by Maintaining Intestinal Microbial Homeostasis

Maternal FMT reduces the abundance of p_*Desulfobacterota* in the feces of rats prenatally exposed to arsenic, alleviating the overall state of antenatal arsenic-induced inflammation and impairing the integrity of the intestinal barrier and blood–brain barrier by reestablishing normal intestinal flora [45]. Moreover, g_*Akkermansia*’s membrane protein Amuc_1100 reduced the severity of acute pancreatitis by reducing the infiltration of macrophages and neutrophils, regulating intestinal flora composition and tryptophan metabolism, and reducing the abundance of p_*Desulfobacterota* among intestinal microbes [46,47]. o_*Erysipelotrichales*, f_*Erysipelotrichaceae,* and g_*unidentified_Lachnospiraceae* all belong to the phylum Bacillota. Butyrate effectively reduced the mortality, systemic inflammation, and intestinal barrier destruction caused by acute pancreatitis by reducing the abundance of *Erysipelotrichales* and *Lachnospirales* in mouse models of acute pancreatitis [48]. In a mouse model of nonalcoholic steatohepatitis, disruption of intestinal microbiome homeostasis was accompanied by a steady increase in *Erysipelotrichales* abundance [49]. A high-fat diet exacerbates this condition by increasing the abundance of *Erysipelotrichaceae* [50]. These bacteria are highly prevalent in disease models such as pancreatitis, hepatitis, arthritis, depression, and high-fat diets, and the decreased abundance of these bacteria in the cecum contents of New Zealand rabbits in the fermented straw group may improve the prevention of these diseases and make important contributions to animal health.

An increase in *Rikenellaceae* increases the susceptibility of mice to arthritis [51], and *Rikenellaceae* is positively correlated with the risk of juvenile idiopathic arthritis [52]. *Christensenellaceae* is highly abundant in the intestines of patients with depression [53]. These bacteria are highly prevalent in disease models of arthritis and depression, and the decreased abundance of these bacteria in the cecum contents of New Zealand rabbits in the fermented straw group may be beneficial for the prevention of these diseases and may make important contributions to animal health [54,55].

*Coprobacter* was found to be less abundant in the gut of patients with systemic lupus erythematosus (SLE) [56] and in chicks exposed to chromium VI [57]. Acupuncture increases *Coprobacter* abundance in patients with functional constipation [58]. The abundances of *Bacteroidota* and *Coprobacter* in the cecum of the fermented straw group were significantly greater than those in the control group, indicating that fermented straw increased the abundance of probiotics such as *Coprobacter*, which may play an important role in the fight against harmful heavy metal pollutants, including cadmium. It can also help animals prevent the risk of developing diseases such as constipation and autoimmune diseases.

*Erysipelotrichia* is a harmful bacterium, and its abundance in the intestines of mice with liver and kidney injury induced by cyclophosphamide was significantly reduced [59]. *Corynebacteriales* contain many pathogenic actinobacteria such as *Corynebacterium graminis* and *Mycobacterium tuberculosis* [60]. Fermented straw promoted the growth of beneficial microorganisms in the cecum, reduced the abundance of harmful microorganisms, and was conducive to balancing the homeostasis of cecum microorganisms and reducing the occurrence of diseases.

K21572 (SusD) and K21573 (SusC) participate in the binding and uptake of polysaccharides and are associated with the recognition and presentation of carbohydrates, suggesting that fermented straw increases the abundance of microorganisms associated with the binding and uptake of carbohydrates in the cecum [61]. The K00059 (FabG) protein, which is involved in bacterial fatty acid synthesis, plays a role in the second step of the extension cycle of FAS-II [62]. K07636 (PhoR) plays an important role in phosphate homeostasis, and fermented straw increased the abundance of PhoR-related microorganisms in the cecum, indicating that phosphorus in the cecum environment may be limited and that this limited concentration may be related to excessive calcium content [63,64]. In the cecum of New Zealand rabbits supplemented with fermented straw, the combination and uptake of carbohydrates, fatty acid synthesis, and the abundance of phosphate homeostasis-related microorganisms increase, which helps New Zealand rabbits digest and absorb more fermented straw and synthesize short-chain fatty acids for energy. Phosphate homeostasis-related microorganisms play a vital role in coping with excessive calcium in the body.

## 5. Conclusions

In conclusion, replacing corn with fermented corn stalk in New Zealand rabbits can significantly increase average daily gain and average daily feed intake and tends to decrease the feed-to-gain ratio, with no negative effect on growth performance. In addition, fermented corn straw can increase microbial enzyme activity in the cecum, reduce the diarrhea index and diarrhea incidence of weaned rabbits, and improve cecum microflora and metabolites and other probiotic effects. This study provides a reference for the application of fermented corn stalks in other monogastric animals such as pigs.

## Figures and Tables

**Table 1:** Composition and nutritional level of experimental diet (%, air dry basis).

Item	Treatments			
	C100	FS50	FS100	DS50
Raw material and total (%, air dry basis)				
Corn	12	6	0	6
Fermented straw	0	6	12	0
Dry straw	0	0	0	6
Wheat flour	15	15	15	15
Wheat bran	24	24	24	24
Soybean meal (43%)	10	11.02	12.05	11.04
Rapeseed meal	2	2	2	2
Alfalfa meal	22	22	22	22
Soybean oil	2	2	2	2
Chaff (chaff powder)	12	11.28	10.25	10.96
Lysine hydrochloride	0.06	0.06	0.06	0.06
Threonine	0.06	0.06	0.06	0.06
Stone powder	0.3	0	0	0.3
Feed additive premix ^a^	0.58	0.58	0.58	0.58
Total	100	100	100	100
Nutrition level (%, air dry basis)				
DM (%) ^b^	88.4	88.9	88.1	90.8
OM (%) ^b^	92.1	91.4	90.3	91.5
CP (%) ^b^	15.8	15.4	15.6	15.4
CF (%) ^b^	25.8	29.1	29.2	27.8
NDF (%) ^b^	47	48.2	48.1	48.1
ADF (%) ^b^	26.3	28	28.7	27.8
ADL (%) ^b^	4.7	4.6	4.1	4.2
AIA (%) ^b^	3	3.42	3.85	3.48
Ca (%) ^b^	0.5	0.7	1	0.5
P (%) ^b^	0.6	0.5	0.5	0.6
Lys (%) ^c^	0.7	0.8	0.8	0.8
Met + Cys (%) ^c^	0.6	0.6	0.6	0.6
Threonine (%) ^c^	0.6	0.6	0.7	0.6
DE (MJ/kg) ^c^	10.7	10.5	10.2	10.4

C100, 12% corn. FS50, 6% corn + 6% fermented straw. FS100, 12% fermented straw. DS50, 6% corn + 6% dry straw. DM, dry matter. OM, organic matter. CF, crude fiber. NDF, neutral detergent fiber. ADF, acid detergent fiber. ADL, acid detergent lignin. CP, crude protein. SDF, soluble dietary fiber. Lys, lysine. Met, methionine. Cys, cystine. DE, digestive energy. AIA, acid insoluble ash. ^a^ The premix provided the following per kg of diet: VA 6000 IU, VD3 1200 IU, VE 50 IU, VK3 2.4 mg, biotin 240 μg, choline 100 mg, pyridoxine 1.8 mg, riboflavin 3.6 mg, VB12 12.5 μg, nicotinamide 20 mg, pantothenic acid 12.5 mg, Fe 30 mg, Cu 6 mg, Zn 35 mg, Mn 8 mg, Se 0.05 mg, Co 0.3 mg, and I 0.4 mg. ^b^ The measured values. ^c^ The calculated values.

**Table 2:** Effect of fermented corn straw as a substitute for corn in feed on the growth performance and total intestinal apparent digestibility of New Zealand rabbits.

Item	Experimental Diet				SEM	*p*-Value
	C100	FS50	FS100	DS50		
Growth performance						
Initial weight (g)	1122	1138	1148	1132	5.3	0.373
Final weight (g)	2067 ^b^	2089 ^b^	2237 ^a^	2071 ^b^	19.69	0.004
Average daily gain (g)	26.7 ^b^	27.2 ^b^	31.5 ^a^	26.8 ^b^	0.54	0.002
Average daily feed intake (g)	114.8 ^b^	118.2 ^b^	129.1 ^a^	117.6 ^b^	1.23	<0.001
Feed-to-weight ratio (F/G)	4.35	4.51	4.17	4.49	0.06	0.112
Total intestinal apparent digestibility						
DM (%, Air dry basis)	56.1 ^a^	52.6 ^c^	51.4 ^d^	54.9 ^b^	0.562	<0.001
CP (%, Air dry basis)	79.2 ^c^	80.8 ^ab^	79.8 ^bc^	81.9 ^a^	0.354	0.003
CF (%, Air dry basis)	17.24	19.09	20.42	19.1	0.933	0.496
NDF (%, Air dry basis)	25.5 ^a^	22.8 ^b^	22.8 ^b^	21.52 ^b^	0.669	0.001
ADF (%, Air dry basis)	12.2 ^b^	9.9 ^b^	11.3 ^b^	16.5 ^a^	0.822	0.004
ADL (%, Air dry basis)	5.4	1.5	1.4	2	0.848	0.12

C100, 12% corn. FS50, 6% corn + 6% fermented straw. FS100, 12% fermented straw. DS50, 6% corn + 6% dry straw. DM, dry matter. CP, crude protein. CF, crude fiber. NDF, neutral detergent fiber. ADF, acid detergent fiber. ADL, acid detergent lignin. ^a–c^ Means within a row with different superscripts are significantly different (*p* < 0.05). Significance testing was carried out using Duncan’s test (*p* < 0.05). Experiments were performed in biological triplicate, with growth performance data collected from 30 rabbits for each repeat and digestibility data from 12 rabbits per group.

## Data Availability

The original contributions presented in this study are included in the article/Supplementary Material. Further inquiries can be directed to the corresponding authors.

## Supplementary Materials

The following supporting information can be downloaded at https://www.mdpi.com/article/10.3390/ani15121737/s1: Table S1: Diarrhea score standard table; Table S2: New Zealand rabbits’ specific primer sequences used for RT-PCR; Table S3: Chemical composition of straw before and after fermentation; Table S4: Hydrolyzed amino acid composition in fermented straw; Table S5: Immune organ index (6 rabbits/group); Table S6: Statistical table of metabolites in cecal content identification; Table S7: FS100_vs_C100. Mantel-test.spearman.single_trait; Table S8: FS100_vs_C100.family.spearman_correlation.filtered; Table S9: FS100_vs_C100.genus.spearman_correlation.filtered; Figure S1: Process characterization of fermented straw; Figure S2: Identifying the mRNA expression of genes associated with glucose metabolism and lipid metabolism in the muscle of New Zealand rabbits; Figure S3. Physiological and morphological responses to fermented corn straw in New Zealand rabbits; Figure S4: Identifying the mRNA expression of genes associated with nutrition transport in the mucosa of the jejunum and ileum; Figure S5: Effects of fermented corn straw on the expression of genes related to intestinal mucosal barrier in New Zealand rabbits; Figure S6: Metabolome analysis of serum and cecal contents; Figure S7: Tax4Fun2 functional annotation clustering heat map and Anosim differences based on ASV; Figure S8: Microbiome analysis of cecum contents and combined analysis of microbiome and metabolome; Figure S9: Statistical map of Metastats significance difference between species based on ASV; Figure S10: Multiomics analysis of serum metabolome, cecal content metabolome, and cecal content microbiome.

## Abbreviations

The following abbreviations are used in this manuscript:

ADF	Acid detergent fiber
ADL	Acid detergent lignin
AIA	Acid insoluble ash
ALB	Albumin
ALT	Alanine aminotransferase
AOD	Average optical density
AST	Aspartate aminotransferase
ASV	Amplicon sequence variant
CD	Crypt depth
CF	Crude fiber
CP	Crude protein
Glu	Glucose
*IL-10*	Interleukin 10
*IL-12*	Interleukin 12
*IL-1β*	Interleukin 1 beta
IOD	Integrated optical density
*LOC103348994*	NADH dehydrogenase [ubiquinone] 1 beta subcomplex subunit 1
*LOC108177067*	NADH dehydrogenase 1 alpha subcomplex subunit 1-like
*MTUS2*	Microtubule associated scaffold protein 2
*MUC2*	Mucin-2
NDF	Neutral detergent fiber
*NDUFA13*	NADH: ubiquinone oxidoreductase subunit A13
*NDUFA6*	NADH: ubiquinone oxidoreductase subunit A6
PAS	Periodic acid–Schiff
SCFA	Short-chain fatty acid
SCFM	Synthetic cystic fibrosis medium
*SLC2A2*	Solute carrier family 2 member 2
*SLC7A5*	Solute carrier family 7 member 5
TC	Total cholesterol
TG	Glyceryl tridodecanoate
*TGF-β1*	Transforming growth factor beta-1
*TJP1*	ZO1 tight junction protein
*TLR2*	Toll-like receptor 2
*TLR4*	Toll-like receptor 4
*TNF*	Tumor necrosis factor
TP	Total protein
VH	Villi height
