# Supplementary material for: The Effects of Pretreated and Fermented Corn Stalks on Growth Performance, Nutrient Digestion, Intestinal Structure and Function, and Immune Function in New Zealand Rabbits
Source: Animals (Basel). 2025 Jun 12;15(12):1737. doi: 10.3390/ani15121737 (PMC12189150; doi:10.3390/ani15121737)
Supplement: Supplementary file 1 [file animals-15-01737-s001.zip › animals-3621968 -Supplementary figures.pdf]

Supporting information to

Evaluation of fermented corn straw as an alternative ingredient to corn for rabbits

Xuying Jia [1], Yaohao Dun [1], Guoqi Xiang [1], Shuai Wang [2], Heng Zhang [1], Wen Zhou [3], Yingjun Li [1,*] and Yunxiang Liang [1,4,*]

[1] National Key Laboratory of Agricultural Microbiology, College of Life Science and Technology, Huazhong Agricultural University, Wuhan 430070, China; jiaxy@webmail.hzau.edu.cn (X.J.); dunyaohao@mail.hzau.edu.cn (Y.D.)

[2] Department of Animal Nutrition and Feed Science, College of Animal Science and Technology, Huazhong Agricultural University, Wuhan 430070, China

[3] Green Chemical Reaction Engineering, Engineering and Technology Institute Groningen (ENTEG), University of Groningen, Nijenborgh 4, 9747 AG Groningen, The Netherlands

[4] Cooperative Innovation Center of Industrial Fermentation (Ministry of Education & Hubei Province), Hubei University of Technology, Wuhan 430068, China

* Correspondence: yingjun@mail.hzau.edu.cn (Y.L.); liangyunxiang@mail.hzau.edu.cn (Y.L.)

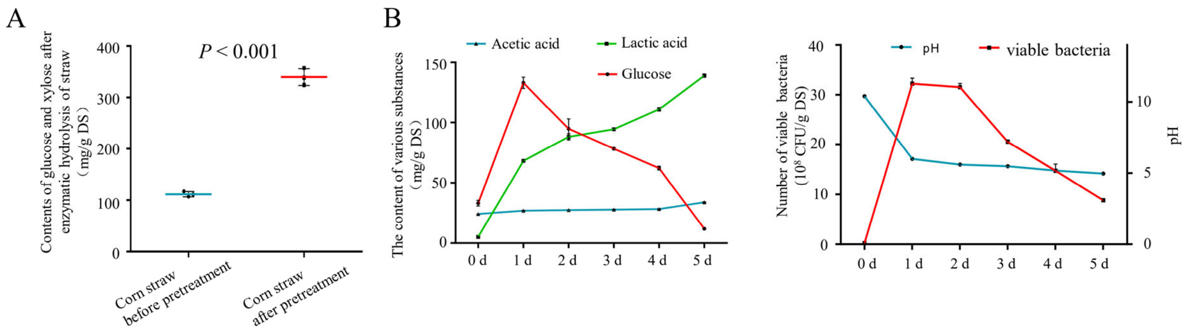

Figure S1. Process characterization of fermented straw. (A) Determination of Sugar content after enzymatic hydrolysis of straw by HPLC. The data represent three independent experiments. Student's t-test was used to analyze statistical significance. DS, dry straw. Experiments were performed in biological triplicate. Student's t-test was used to analyze statistical significance. (B) Changes of glucose, organic acid, pH and viable bacteria in the first 5 days of fermentation.

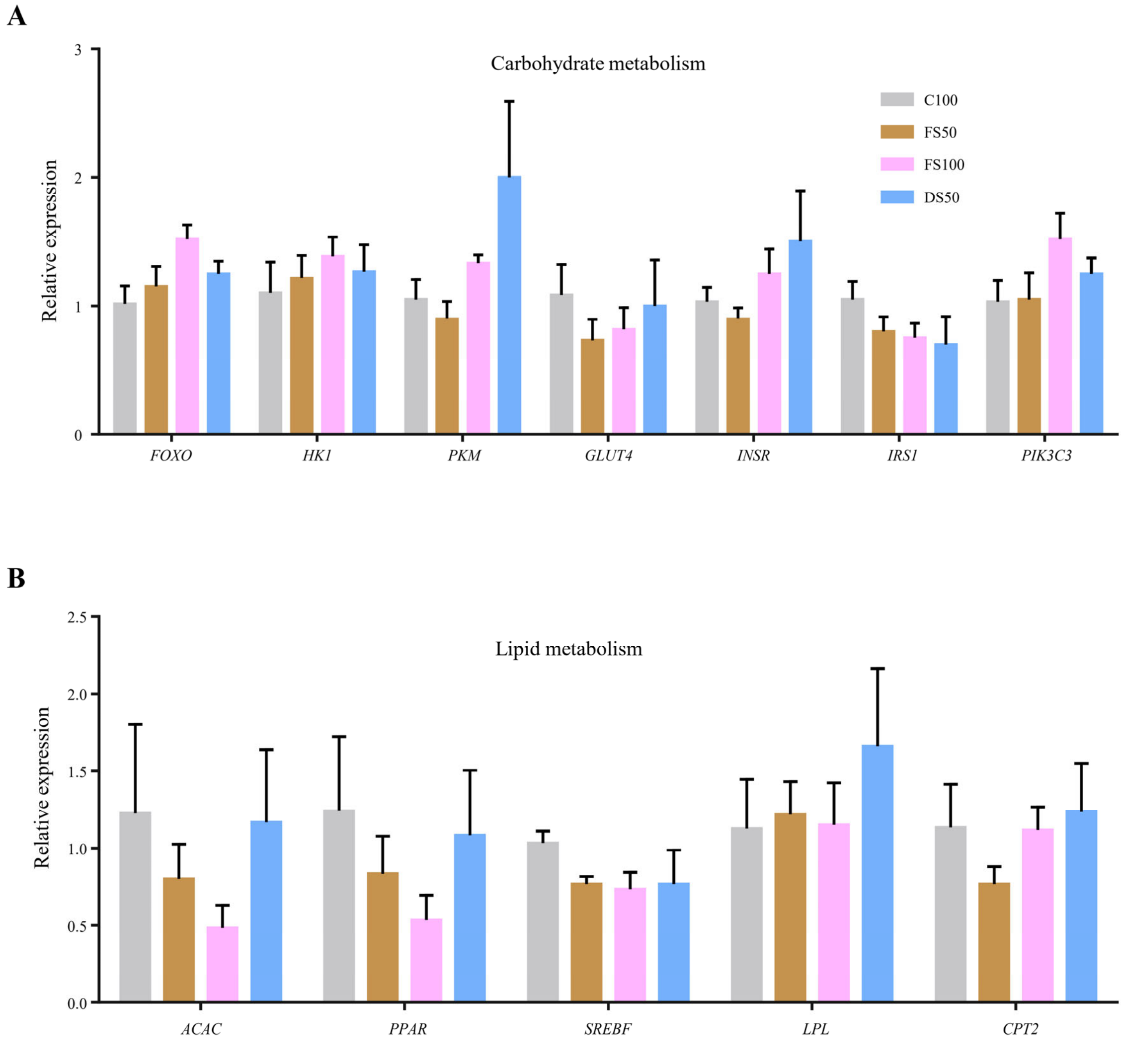

Figure S2. Identifying the mRNA expression of genes associated to glucose metabolism and lipid metabolism in muscle of New Zealand Rabbits. (A) Detection of glucose Metabolism related Gene expression in muscle by fermented corn straw. (B) Detection of Lipid Metabolism related Gene expression in muscle by fermented corn straw.

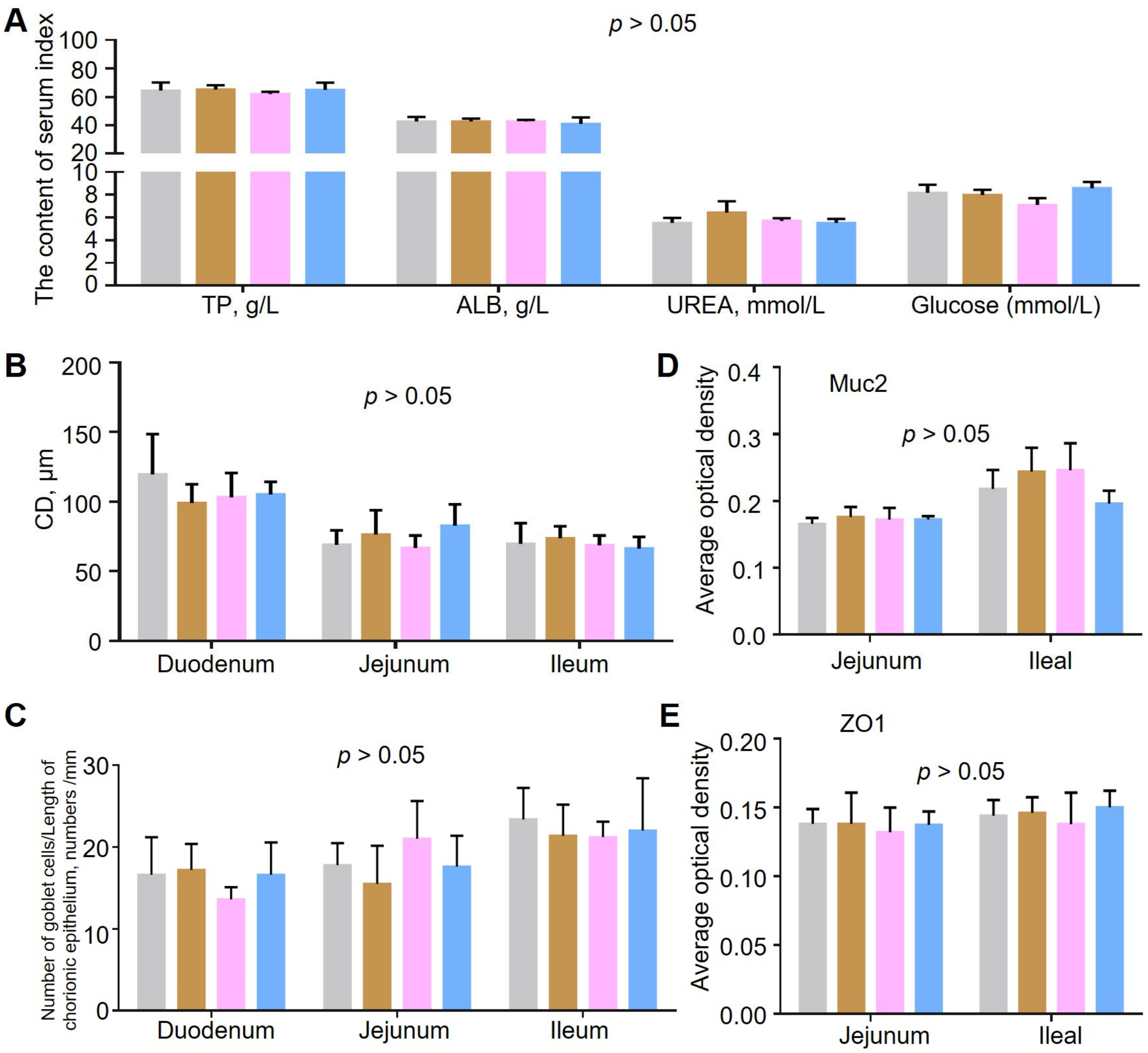

Figure S3. Physiological and morphological responses to fermented corn straw in New Zealand rabbits. (A) Biochemical parameters in serum. TP, total protein. ALB, albumin. Significance testing was carried out using Duncan's test ($P < 0.05$). Experiments were performed in biological triplicate, with 6 New Zealand rabbits for each repeat. (B) Crypt depth of duodenum, jejunum and ileum. CD, crypt depth. The bars represent the means standard errors of each group (n = 6). Means within a row with different superscripts significantly different. (C) The number of goblet cells in duodenum, jejunum and ileum. (D) Statistics of average optical density of Muc2 immunohistochemical detection in jejunum and ileum sections. (E) Statistics of average optical density of ZO1 immunohistochemical detection in jejunum and ileum sections.

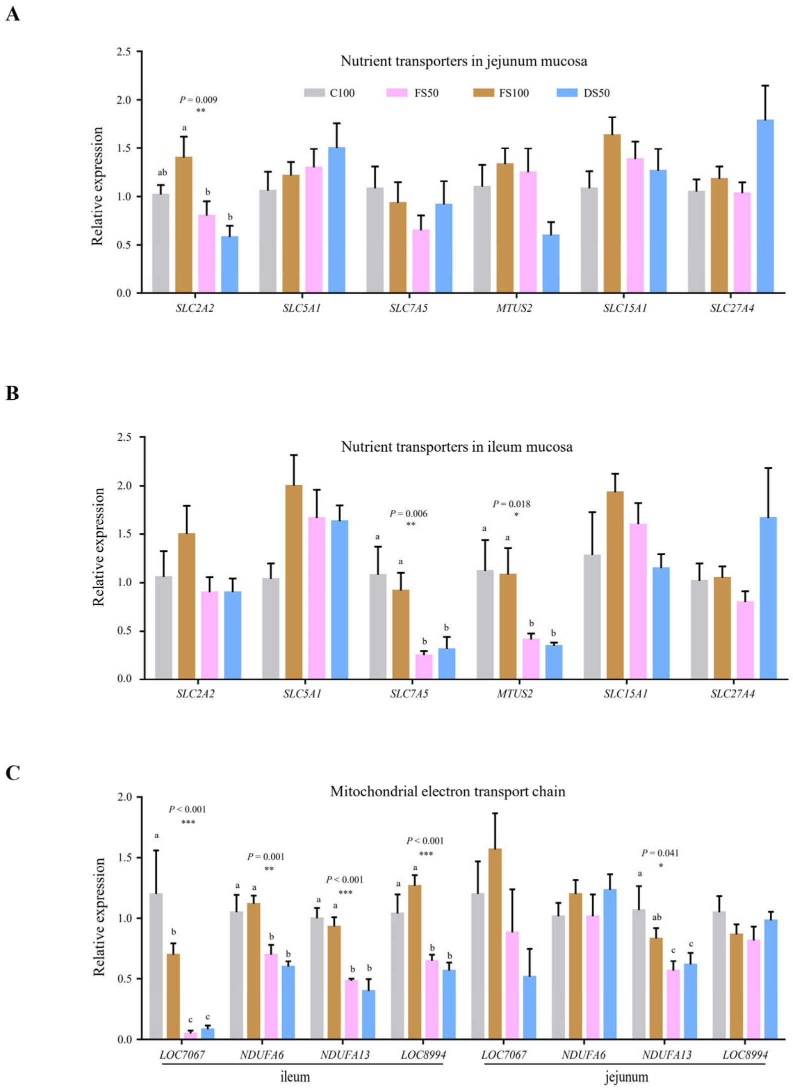

Figure S4. Identifying the mRNA expression of genes associated to nutrition transport in the mucosa of the jejunum and ileum. (A) Detection of gene expression related to glucose transporter, amino acid transporter and fatty acid transporter injejunal mucosa. *SLC2A2* and *SLC5A1* are glucose transporter related gene. *SLC7A5*, *MTUS2* and *SLC15A1* are amino acid transporter related gene. *SLC27A4* is fatty acid transporter related gene. (B) Detection of gene expression related to glucose transporter, amino acid transporter and fatty acid transporter in ileal mucosa. (C) Detection of gene expression related to electron transfer chain in jejunal and ileal mucosa.

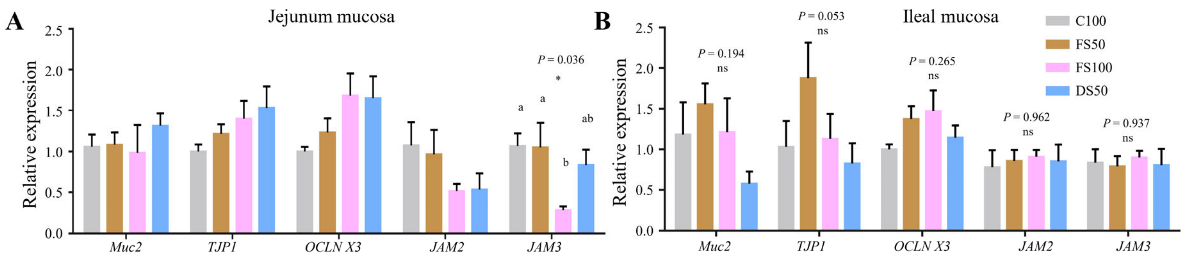

Figure S5. Effects of fermented corn straw on the expression of genes related to intestinal mucosal barrier in New Zealand rabbits. (A)Detection of intestinal barrier related gene expression in jejunal mucosa. (B)Detection of intestinal barrier related gene expression in ileal mucosa.

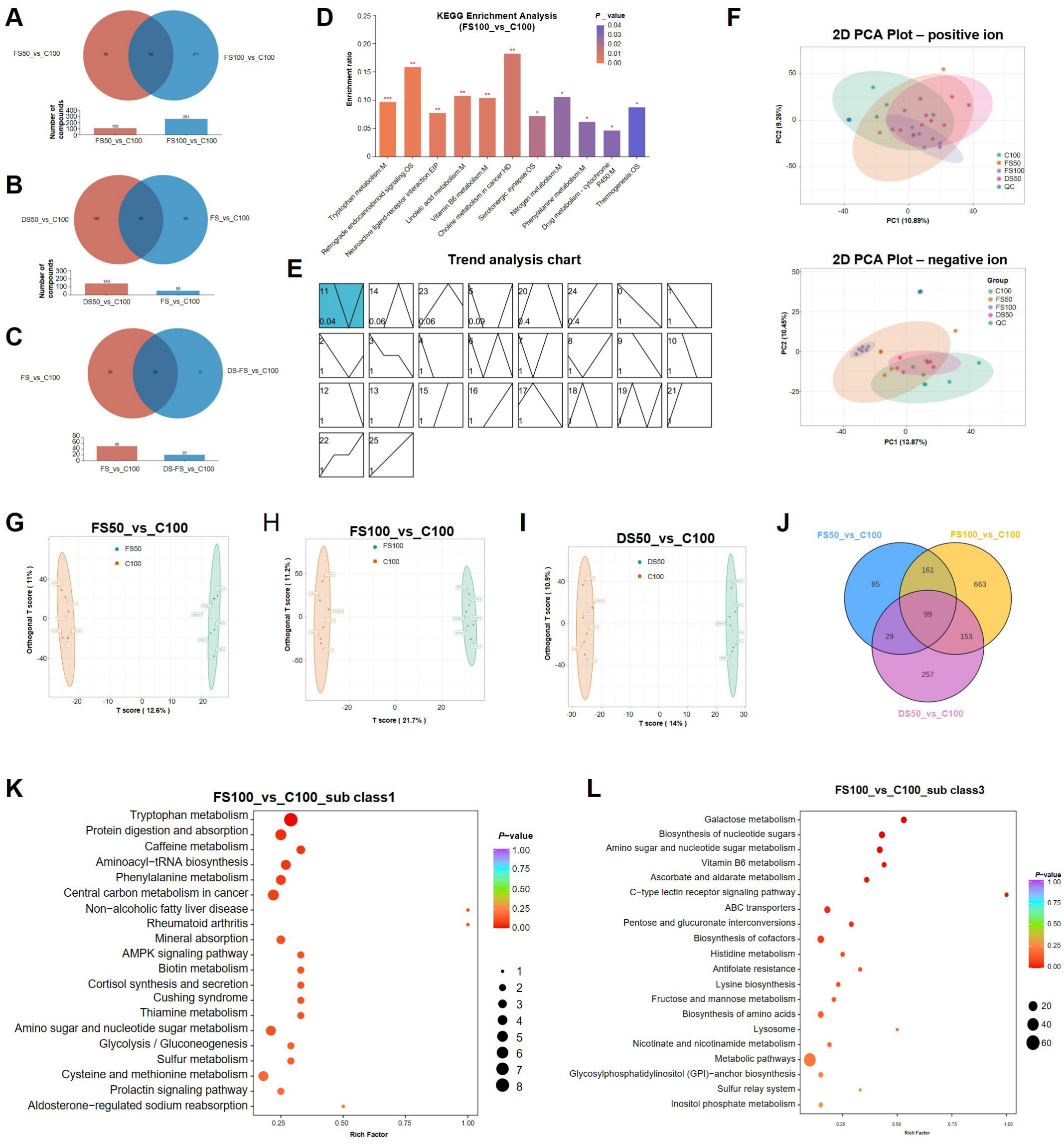

Figure S6. Metabolome analysis of serum and cecal contents. (A) Venn diagrams of differential metabolites between FS50 and FS100 and C100, respectively. (B) Venn diagrams of Metabolic set DS50_vs_C100 and Metabolic set FS50_FS100_C100. (C) Venn diagrams of Metabolic set FS50_FS100_C100 and Metabolic set DS50_vs_C100. (D) KEGG Pathway enrichment Analysis Map of metabolic set VIP_FS100_C100. The Abscissa represents the difference abundance score (DA-Score) and the ordinate indicates the name of the KEGG metabolic pathway. (E) Time Series expression trend Analysis of metabolic set FS100_vs_C100. (F) PCA score chart of each sample quality spectrum data. PC1 represents the first principal component, PC2 represents the second principal component, PC3 represents the third principal component, and percentage represents the interpretation rate of this principal component to the data set. (G) OPLS-DA analysis model diagram of FS50_vs_C100. The horizontal coordinate represents the prediction component score, and the horizontal coordinate direction can show the difference between groups. (H) OPLS-DA analysis model diagram of FS100_vs_C100. (I) OPLS-DA analysis model diagram of DS50_vs_C100. (J) Venn diagram of differences among groups. DS50_vs_C100, representing the differential metabolite between DS50 and C100 groups. FS100_vs_C100 represents the differential metabolite between FS100 and C100 groups. DS50_vs_C100 represents the differential metabolite between DS50 and C100 groups. (K) KEGG enrichment diagram of subclass1. (L) KEGG enrichment diagram subclass 3.

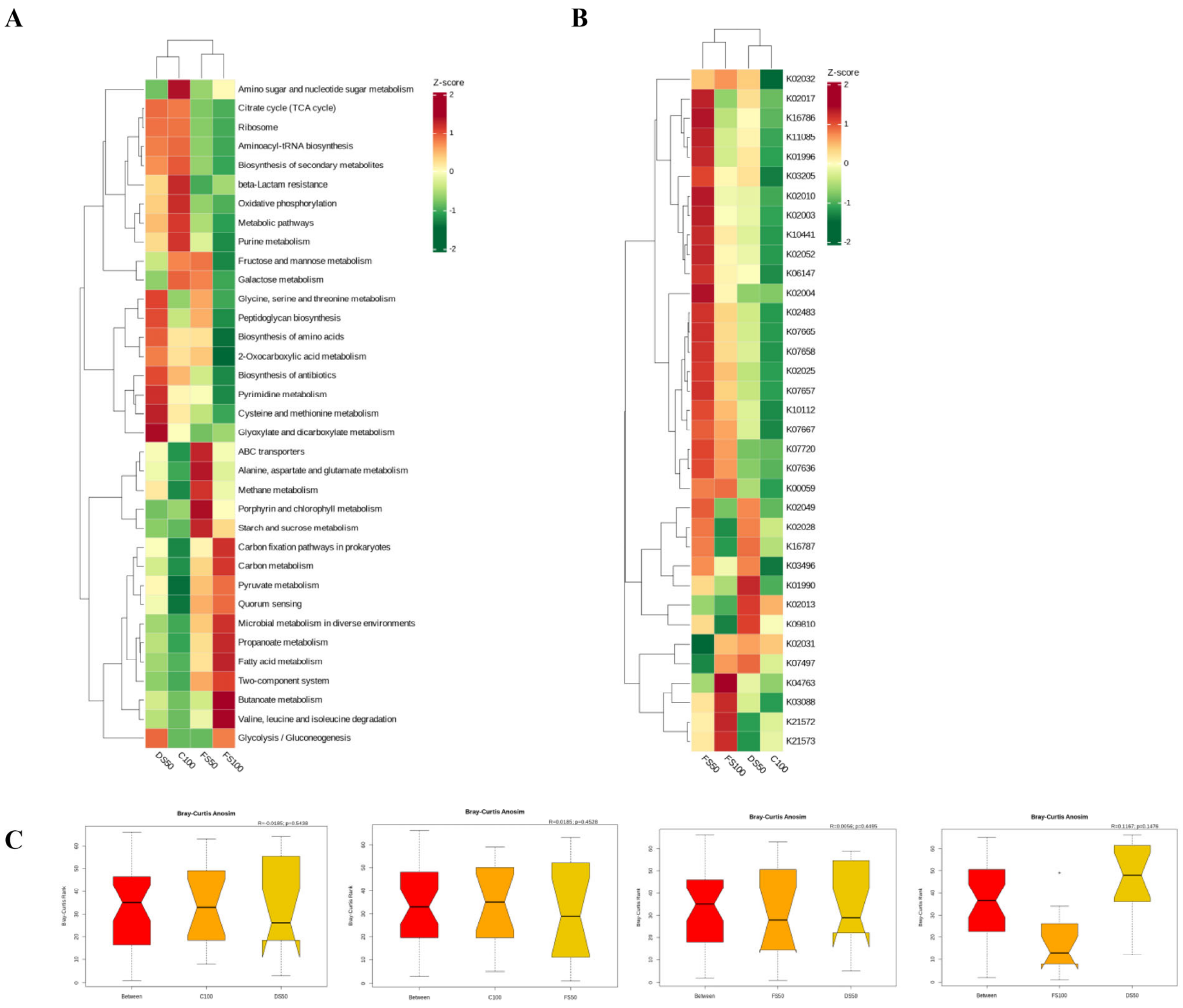

Figure S7. Tax4Fun2 functional annotation clustering heat map and Anosim differences based on ASV. (A) Level1 function annotation clustering heat map. According to the sum of the abundance of all samples in the database function annotations, the top 35 functions with the highest abundance and their abundance information in each sample were selected to draw a heat map, and clustering was carried out from the level of functional differences. (B) Levelk function annotation clustering heat map. (C) ASV based analysis of Anosim differences between groups. The ordinate is the rank of the distance Between the samples, the abscissa: Between is the result between the two groups, and the other two are the result within their respective groups.

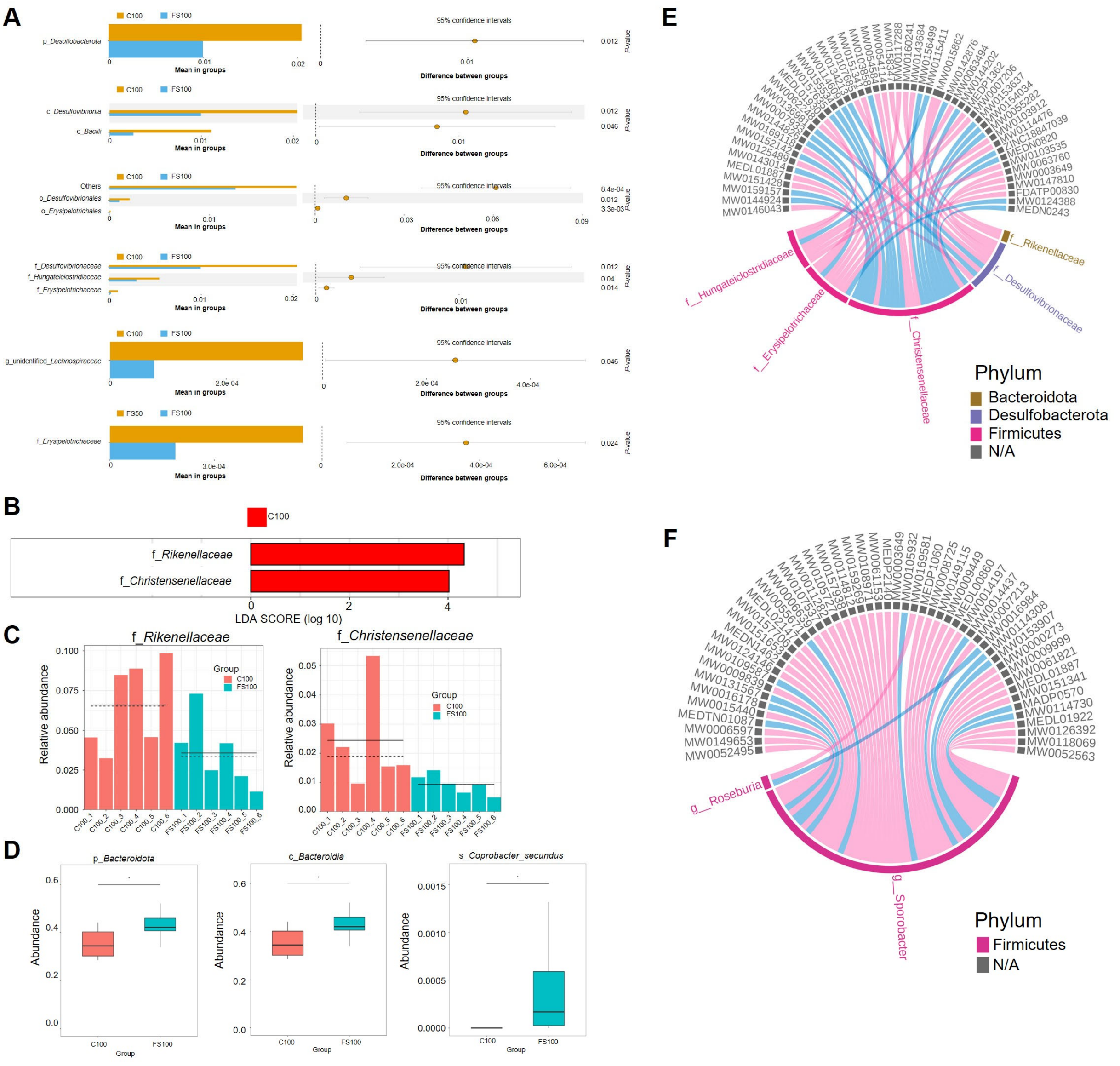

Figure S8. Microbiome analysis of cecum contents and combined analysis of microbiome and metabolome. (A) T-test analysis of different species between groups. (B) Histogram of LDA value distribution obtained from analysis of LEfSe (LDA Effect Size) of different species between groups. (C) LDA Effect Size (LEfSe) analysis of the biomarkers with statistical differences in abundance in different groups. (D) Metastats analysis of differential species between groups (differential species with up-regulated abundance in FS100 group). "*" indicates a significant difference between the two groups ($P < 0.05$). (E-F) Spearman correlation chord diagram of FS100_vs_C100 differential microorganisms and differential metabolites.

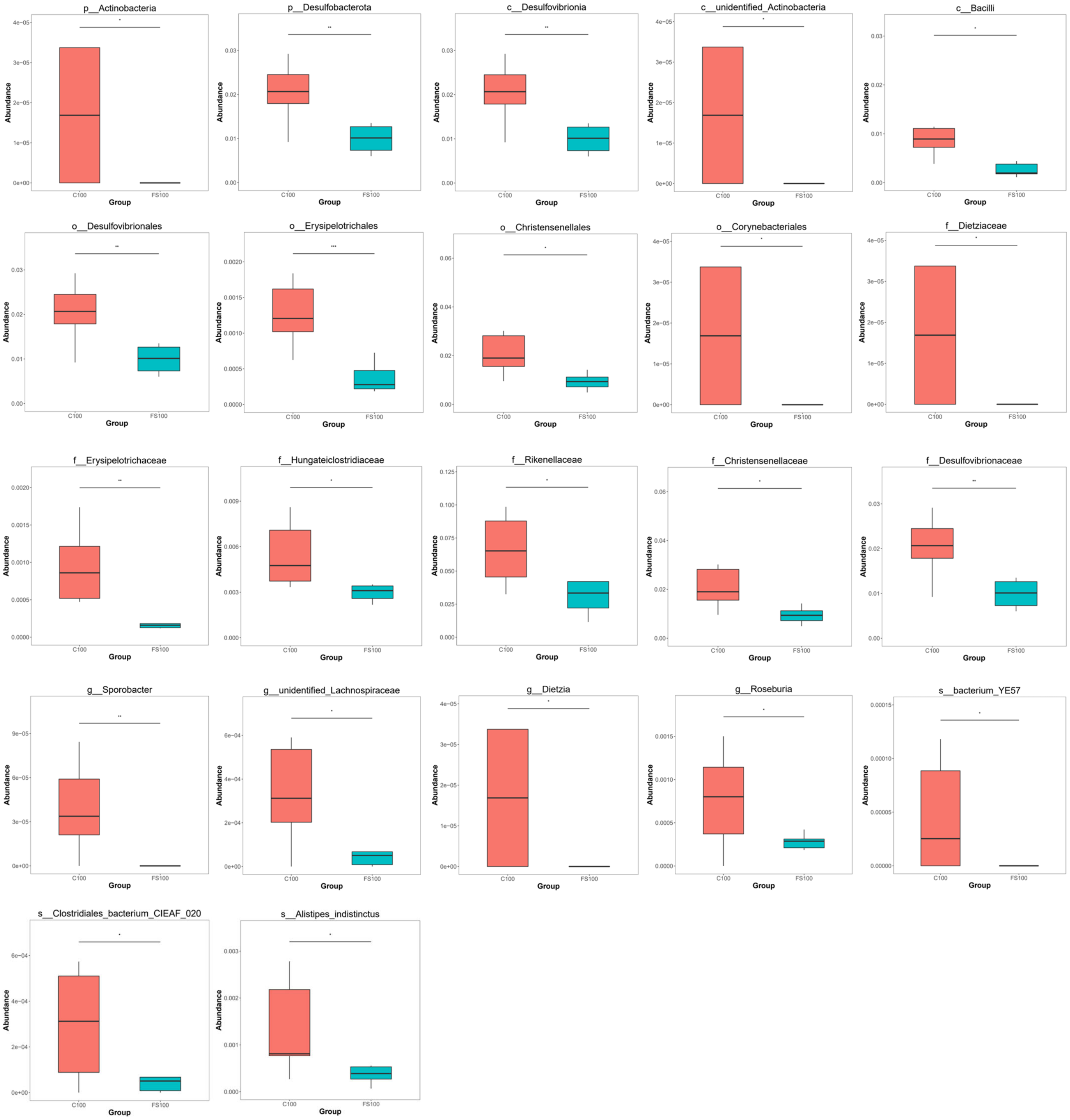

Figure S9. Statistical map of Metastats significance difference between species based on ASV. Metastats analysis of differential species between groups (differential species with down-regulated abundance in FS100 group). The horizontal axis is sample grouping; The vertical axis is the relative abundance of the corresponding species. The horizontal line represents the two groups with significant differences, and none indicates that there is no difference between the two groups. "*" indicates a significant difference between the two groups (P-value < 0.05), and "**" indicates a significant difference between the two groups (P-value < 0.01).

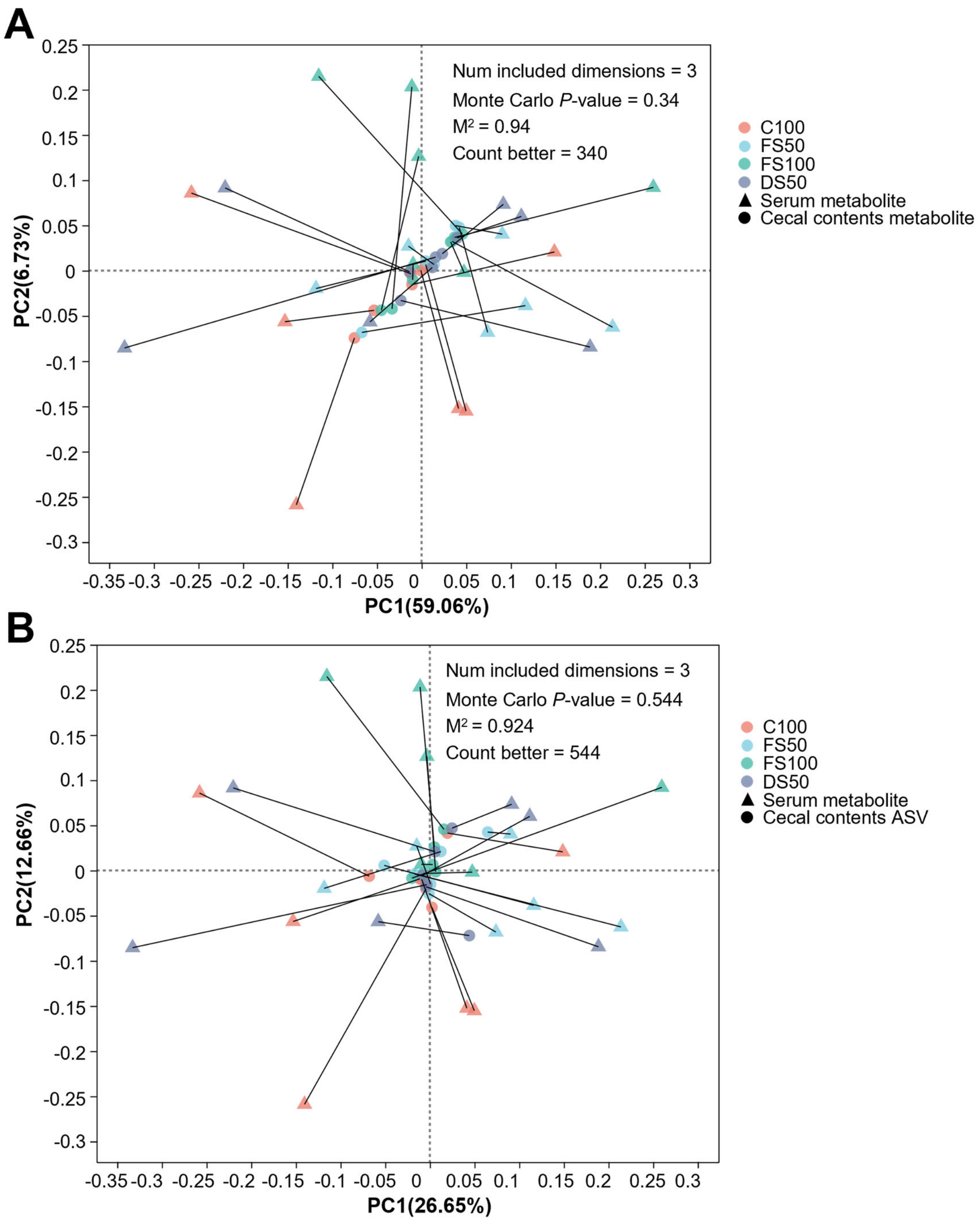

Figure S10. Multiomics analysis of serum metabolome, cecal content metabolome and cecal content microbiome. (A) Procrustes Analysis between serum metabolome and cecal content metabolome. (B) Procrustes Analysis between serum metabolome and ASV diversity of cecal contents. Monte Carlo *P*-value: indicates the *P*-value generated by Monte Carlo simulation. $P < 0.01$ indicated that the trend of association characteristics and metabolite expression was significantly consistent among different groups; $P < 0.05$ indicated that the trend of association characteristics and metabolite expression was significantly consistent among different groups; *P* >= 0.05 indicated that the trend of association characteristics and metabolite expression was not significantly different among different groups. Countbetter: The minimum number of simulations in which the error value is less than or equal to the actual error value in the monte Carlo simulation; M^2: sum of squares of deviation analyzed by Procrustes. A smaller value indicates a higher degree of association between two data sets. The M^2 statistic and its significance test *P* -value provide an overall measure of consistency between two datasets.
